# Evidence Relating to Environmental Noise Exposure and Annoyance, Sleep Disturbance, Cardio-Vascular and Metabolic Health Outcomes in the Context of IGCB (N): A Scoping Review of New Evidence

**DOI:** 10.3390/ijerph17093016

**Published:** 2020-04-26

**Authors:** Irene van Kamp, Sendrick Simon, Hilary Notley, Christos Baliatsas, Elise van Kempen

**Affiliations:** 1 National Institute for Public Health and the Environment, 3721 MA Bilthoven, The Netherlands; Sendrick.simon@rivm.nl (S.S.); Elise.van.kempen@rivm.nl (E.v.K.); 2 UK Department for the Environment, Food and Rural Affairs (Defra), Ground Floor, Seacole Building, 2 Marsham Street, London SW1P 4DF, UK; noise@defra.gov.uk; 3 Netherlands institute for health services research, 3513 CR Utrecht, The Netherlands; c.baliatsas@nivel.nl

**Keywords:** noise, health, WHO environmental noise guidelines for the European region, annoyance, sleep disturbance, cardiovascular disease and metabolic disorders

## Abstract

WHO published the Environmental Noise Guidelines for the European Region in 2018, based on seven systematic reviews including studies published between 2000 and 2014. Since then, new studies were published. At the request of the UK Department for Environment, Food and Rural Affairs (DEFRA), a review on annoyance, sleep disturbance, cardiovascular and metabolic effects in relation to environmental noise was prepared. The aim was to advise the Interdepartmental Group on Costs and Benefits Noise Subject Group (IGCB(N)) whether this new evidence warrants an update of their recommendations. Four databases for observational studies were screened and data were extracted on design, type and measurements of exposures and outcomes and confounders and their associations. The quality of the studies was indirectly assessed for cardiovascular and metabolic effects by only including studies with a case control or cohort design. For studies on annoyance and sleep disturbance, the risk of bias was expressed in exposure misclassification, selective participation and confounding. The update yielded 87 papers, pertaining to 108 new studies of which 40 new studies were on annoyance, 42 on sleep disturbance and 26 concerning cardiovascular and metabolic effects. The number, size and quality of the new studies suggest new meta-analyses could be undertaken over the sources and effects included in the WHO reviews.

## 1. Introduction

Unlike several other environmental stressors, environmental noise, especially in the urban environment, is still increasing. Evidence supports an association between higher levels of environmental noise and various adverse health effects, such as cardiovascular diseases, metabolic effects, sleep disturbance, annoyance and impacts on cognitive development in schoolchildren (7–11 years old). Noise from transport ranks among the environmental stressors with the highest public health impact and was identified as the second most significant environmental cause of ill health in Western Europe, the first being air pollution from fine particulate matter [1]. In the European area, approximately 16,600 cases of premature death are associated with environmental noise. Additionally, around 32 million adults are estimated to suffer annoyance and over 13 million adults suffer from sleep disturbance [1].

In 2018, the Environmental Noise Guidelines for the European Region of the World Health Organization (WHO) were published [2]. Underlying the Guidelines were seven systematic evidence reviews on adverse birth effects, hearing loss and tinnitus, cognitive effects, mental health, annoyance, sleep disturbance, cardiovascular and metabolic effects [3,4,5,6,7,8,9]. These reviews were published in a special issue of the International Journal of Environmental Research and Public Health (IJERPH) in the period between August 2017 and October 2018. While the earlier Noise Guidelines were primarily focused on transportation noise, the new Guidelines include wind turbine noise and leisure noise [10]. This was a consequence of the need for guidelines for sources other than transport, as was emphasised at the European Ministerial Conference at Parma in 2010 (WHO Regional Office for Europe, 2010). Industrial noise, neighbourhood noise and low frequency noise were not included in the new Guidelines because of a lack of evidence, heterogeneity of noise caused by industrial noise source and their local variation. 

Based on the evidence review of Basner and McGuire, we know that there is evidence of sufficient strength for self-reported and objective indicators of sleep disturbance due to environmental noise [3]. Studies investigating the association between noise and sleep disturbance are usually based on the percentage of highly sleep disturbed (%HSD) as measures on a semi standard question referring to the noise source [3]. Objective measures include motility data and cortical awakenings. As part of their review, Basner and McGuire derived Exposure Effect relations (EErs) for the percentage of highly sleep disturbed people related to L_night_ for aircraft, rail and road traffic-related noise. For wind turbine noise, the evidence is only emerging and no EEr is available yet. The newly derived source-specific EErs for sleep disturbance show considerable variation within and between studies. This indicates that deviations in the %HSD can occur at national/regional/municipal level as compared to %HSD calculated using the generalised EErs described in the review. The heterogeneity of the studies thus limits the value of the generic EErs. 

There is also evidence of sufficient strength for environmental noise annoyance [4]. Studies investigating the association between noise and annoyance are based the percentage of highly annoyed (%HA) on a standard survey question (ISO/TS 15666:2003, https://www.iso.org/standard/28630.html) that refers to the noise source in the question. In principle, EErs for the %HA related to L_den_ are available for the noise sources aircraft, railway, and road traffic. The latest ones were derived by Guski et al. For wind turbine noise, the evidence is only emerging and it was not possible to derive a reliable generalised EEr yet. The source-specific EErs for severe annoyance show considerable variation within studies and between studies. This indicates that deviations in the %HA can occur at national/regional/municipal level, as compared to %HA calculated using the generalised EErs. 

There is evidence of enough strength for an association of coronary heart disease (CHD) and stroke (morbidity and mortality) with environmental noise [6,7]. At the time of the WHO review, the most comprehensive evidence was available for road traffic noise and CHD. Only a few studies reported on the association between transportation noise and stroke at the time. End points such as high blood pressure changes in children and hypertension were also considered as health outcomes in the WHO review. However, according to the evidence review, the quality of the evidence supporting an association between traffic noise exposure and hypertension, and blood pressure in children, was considered to be very low, indicating that any estimate of an effect is uncertain. 

Evidence on diabetes and/or obesity was based on a limited number of studies in comparison to, e.g., coronary heart disease [6,7] and it was concluded that, for a comprehensive assessment of the impact of noise exposure on the metabolic system, more and of better quality evidence is needed.

The Guidelines are based on the reviews of scientific literature summarised above, including papers published between 2000 and 2014/2015. Since then, many new publications have emerged, describing results of existing and new studies, that were not part of the WHO reviews. Therefore, the Department for Environment, Food and Rural Affairs (DEFRA) of the UK Government, on behalf of the Interdepartmental Group on Costs and Benefits Noise Subject Group (IGCB(N)), asked the Netherlands Institute for Public Health and the Environment (RIVM to investigate whether the literature reviews, which were prepared in the framework of the WHO Environmental Noise Guidelines, would potentially benefit from an update. Criteria were, therefore, formulated to make a statement about whether the use or need for adaptation of the exposure response relationships and/or risk ratios should be considered by the IGCB(N) for cardiovascular and metabolic effects, annoyance and sleep. In other words, the assignment was to determine how many studies of good quality (per source and outcome) have been published since the end dates covered by the WHO evidence reviews.

To provide evidence-based advice, this paper describes the results of a scoping synthesis of the literature into the effects of environmental noise on health, published between January 2014 and December 2019 in relation to transport and wind turbine noise. The health component focuses on sleep disturbance, annoyance, cardiovascular and metabolic effects.

## 2. Materials and Methods

The scoping synthesis of the new literature consists of two segments. The first segment includes all literature published between January 2014 and June 2019, addressing transport and wind turbine noise in relation to annoyance and sleep disturbance, including some papers of later date (December 2019) published online before print. The second segment consists of literature published between January 2015 and June 2019 about the impact of noise from transport and wind turbines on the cardiovascular and metabolic system. Search terms were identified and discussed by the authors (see Table 1 and Supplementary 1) and followed the protocol of the WHO evidence reviews as much as possible. The bibliographic databases that were utilised included Scopus, MEDLINE, EMBASE and PsycINFO (the latter only for annoyance and sleep disturbance). Within each database and depending on the segment, we screened all Dutch, English, French and German papers in their respective period.

### 2.1. Study Identification and Selection

**Figure 1:**
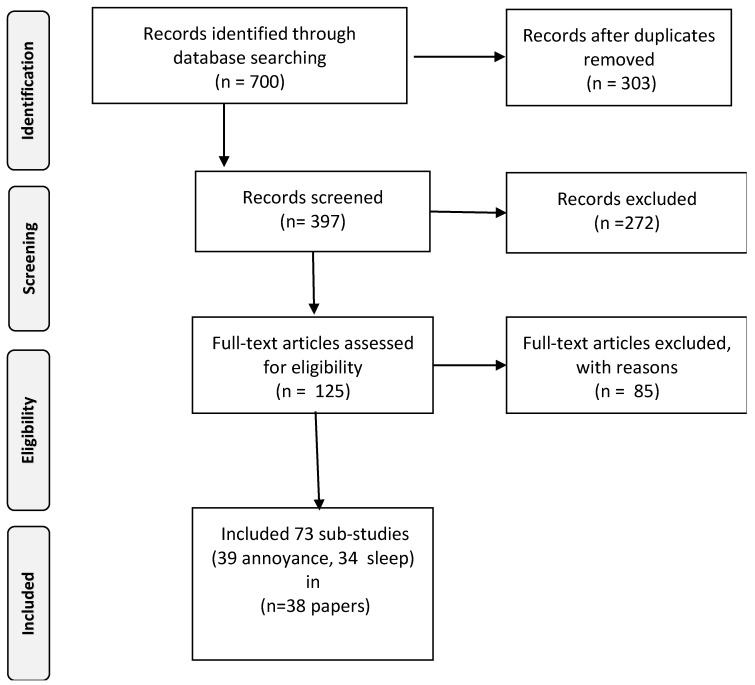
Flowchart outlining the study selection process for annoyance and sleep disturbance.

**Figure 2:**
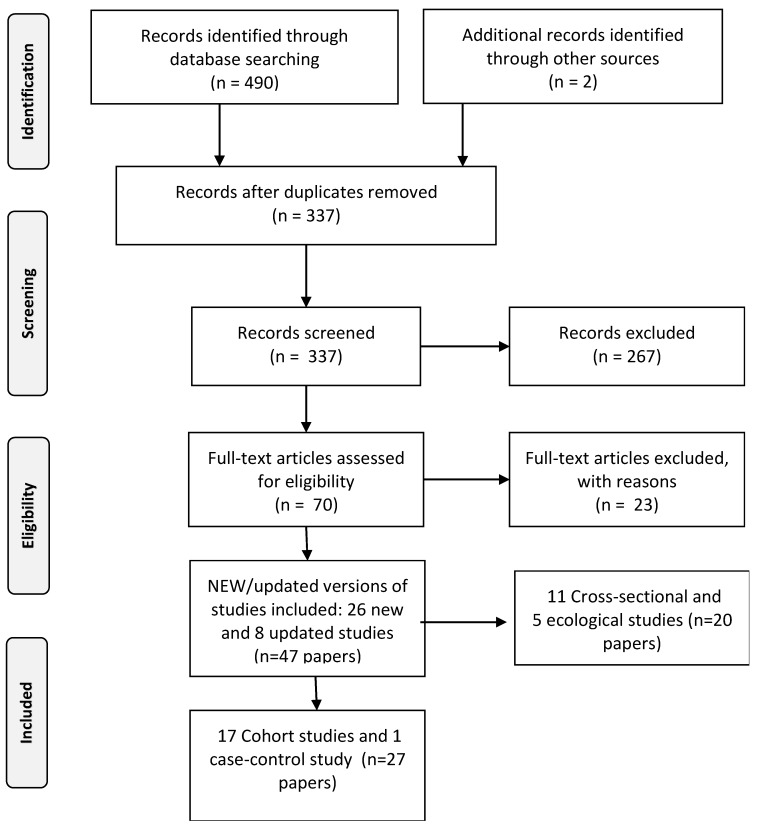
Flowchart outlining the study selection process for cardiovascular and metabolic effects.

The review process consisted of three major steps: literature searching, title and abstract screening and full text screening (see for the full profiles Supplementary 1). During the first phase, 1192 papers were identified by searching the bibliographic databases for further review; 700 for the first segment and 492 for the second segment. During the second phase, the titles and abstract of the selected papers were screened independently by two researchers. This was conducted according to the search protocol as outlined in the research proposal for DEFRA. (Supplementary 1) Papers that clearly did not match the inclusion criteria were hereby excluded. Finally, the full text of the remaining 195 papers (125 for segment I and 70 for segment II) were critically and independently assessed again by two researchers using the same inclusion criteria as for the title and abstract screening, leaving 87 papers to be included in the scoping synthesis; 40 papers for segment I and 47 papers for segment II. The selection process was documented in ample detail by applying a PRISMA-flowchart (see Figure 1 and Figure 2) [11]. Discrepancies during the selection and screening process were solved by discussing and seeking consensus between the researchers. Detailed information about the search terms used for the different outcomes and data sources are presented in Supplementary 1. Table 1 below presents the search profile.

### 2.2. Data Extraction

After reaching consensus, the data were extracted, coded and imported into tables. In the case of disagreement, the evaluators and librarian discussed the options. In principle, the following characteristics of the studies were extracted and coded for each selected study:

- Acronym/Author and Year of Publication;
- Study Design (including sampling strategy);
- Type and source of exposure;
- Characteristics of the population under investigation;
- Exposure source, characterisation (and range);
- Outcome type and ascertainment of the outcome.

For the studies that investigated the association with annoyance and/or sleep disturbance, the following aspects were extracted as well:

- Adjustment for possible confounders;
- Direction and strength of reported effect size [6].

### 2.3. Assessment of Quality and Risk of Bias

Considering time constraints, evaluation of the quality of the studies and risk of bias were dealt with differently for the separate segments of the review.

- For annoyance and sleep disturbance, the study quality was evaluated by two researchers. Thereto, short and user-friendly instruments of the National Institute of Health (NIH) (https://www.nhlbi.nih.gov/health-topics/study-quality-assessment-tools) were used in the assessment. The risk of bias due to exposure misclassification, selective participation and confounding was assessed for the relevant studies as proposed by Grimes and Schulz [12]. The method of rating was broadly based on schemes used by previous systematic reviews [13].
  Risk of bias was categorised as low, medium or high.
- In the segment of the review assessing cardiovascular and metabolic effects, quality evaluation was based on the study design, including only case control or cohort studies. These types of studies are commonly regarded as having high quality.

## 3. Results

### 3.1. Segment I: Environmental Noise in Relation to Sleep Disturbance and Annoyance

Based on our selection criteria we identified 38 new papers [14,15,16,17,18,19,20,21,22,23,24,25,26,27,28,29,30,31,32,33,34,35,36,37,38,39,40,41,42,43,44,45,46,47,48,49,50,51,52] pertaining to 73 observational (sub-) studies on the association between everyday life exposure to aircraft noise (20), road traffic noise (25), rail traffic noise (14) and wind turbine noise (14) in relation to annoyance and/or sleep disturbance. Of the selected studies. 60% was conducted in Europe and 40% elsewhere. Typical for the newly identified studies on annoyance is the geographical spread of the studies including more studies from Asia (eight), South America (one), India (one) and Canada (four) than was the case in the previous (WHO) reviews on annoyance and sleep [3,4]. In 14 of the selected publications, only the association with annoyance was investigated; in 13 publications, the association with only sleep disturbance was investigated. In total, 13 publication described the results of both outcomes; we counted them as separate studies. The results are presented in Supplementary 2, Tables S1.1–S1.9.

#### 3.1.1. Studies Investigating the Impact of Environmental Noise on Sleep Disturbance 

This review yielded thirty-four new (sub) studies investigating the association between environmental noise and sleep disturbance described in 16 papers [36,37,38,39,40,41,42,43,44,45,46,47,48,49,50,51,52]. Fifteen papers described self-reported sleep disturbance as an outcome and three objective measures. According to the PRISMA criteria, the number of studies with sufficient sample size and of good quality for risk of bias evaluation is fair. Overall, the results on sleep disturbance effects are not consistent, primarily due to methodological differences between the studies, nevertheless pointing in the same direction. This pattern is in line with the WHO review [3]. New evidence has been described on the role of the number of events and the L_max_ levels, and it would be interesting to compare the outcomes from the different new studies including the different noise indicators.

#### 3.1.2. Studies Investigating the Impact of Environmental Noise on Annoyance 

The new literature research yielded 39 new (sub-) studies described in 21 papers investigating the association between environmental noise and annoyance [14,15,16,17,18,19,20,21,22,23,24,25,26,27,28,29,30,31,32,33,34,35]. According to the Prisma criteria for risk of bias, the number of studies with a large sample size and of medium to good quality has increased, in particularly for wind turbine noise. Overall, the annoyance outcomes show a similar pattern across noise levels as compared to previous findings in the WHO review [4].

### 3.2. Segment II: Environmental Noise in Relation to Cardiovascular and Metabolic Effect

The forty-seven papers on cardiovascular effect [33,47,53,54,55,56,57,58,59,60,61,62,63,64,65,66,67,68,69,70,71,72,73,74,75,76,77,78,79,80,81,82,83,84,85,86,87] and metabolic effects [88,89,90,91,92,93,94,95,96,97], and cardiovascular effects in children [98], described 34 different observational studies. These studies investigated the association between everyday life exposure to environmental noise in relation to cardiovascular and metabolic effects. Eight of these studies were already included in the WHO evidence review [55,56,60,61,66,67,68,69,75,83] and contained updated and/or additional results, while 26 studies were new [53,54,57,58,59,63,70,71,72,73,76,77,78,79,80,81,82,84,85,86,87,88,89,90,91,92,93,94,95,96,97]. In 27 of the 47 papers, the results of a cohort- or case-control study were presented. All of the outcomes studied in these 27 papers (describing the results of 18 studies) were assigned to one of the six categories: hypertension (10), ischemic heart disease (12), stroke (nine), diabetes (four), (indicators of) obesity (three) and children’s blood pressure (0). Some studies examined more than one outcome. The results are presented in Supplementary 2, Tables S2.1–S2.18.

#### 3.2.1. Studies Investigating the Impact of Environmental Noise on the Incidence of Hypertension

Ten of the evaluated studies investigated the association between noise exposure and the incidence of hypertension [53,54,57,58,59,62,63,64,70,71]. Incidence of hypertension [52,53,54,55,56,57,58,59,60,61,62,63,64,65,66,67,68,69,70,71] was hereby ascertained by the measurement of blood pressure levels and/or by a clinical interview, by means of a question as part of a questionnaire or interview (self-reported) or by means of health registration database. Three studies investigated the effect of exposure to aircraft noise [53,54,58,59,60]; nine investigated the effect of exposure to road traffic noise, two investigated the effect of exposure to rail traffic noise [54,57,58,59] and one investigated the effect of exposure to wind turbine noise [70,71]. Within the evaluated studies, two studies demonstrated a significant positive relationship between aircraft noise and incidence of hypertension. For road traffic noise, five of the nine studies, identified as part of the new literature research, were already included in the WHO evidence review but contained new and/or additional results. For the HUBRO study (also known as the Oslo Health Study), Heinz-Nixdorf Recall (HNR) study and KORA (Cooperative Health Research study), only cross-sectional results were available during the execution of the WHO-evidence review. For the current overview, prospective results were available for these studies. Since the execution of the WHO evidence review, the researchers of the Danish Diet Cancer and Health Cohort (DCH) study published an update of their results. The results of the newly published results of the DCH study hardly changed: still, no association between road traffic noise and the incidence of hypertension.

For rail traffic noise, the two new studies did not find an association between exposure and the incidence of hypertension. For wind turbine noise, the authors of the Danish Wind turbine Cohort Study (DWS) [70,71] concluded that their study does not support an association between wind turbine noise and redemption of antihypertensive medication. 

#### 3.2.2. Studies Investigating the Impact of Noise on Ischemic Heart Disease

Twelve of the evaluated cohort- and case-control studies investigated the association between environmental noise and ischemic heart disease (IHD). [53,72,73,76,77,78,79,80,81,82,84,85] Within these, several studies addressed more than one type of environmental noise. Four studies investigated the impact of aircraft noise [53,57,72,73,76,77], 10 of road traffic noise, [53,58,72,73,76,77,78,79,80,81,82], three of rail traffic noise [58,72,73,76,77] and two were related to wind turbine noise [71,84,85]. The incidence or mortality of IHD was ascertained by means of a clinical interview/anamnesis or by means of a health registration database. 

The association between aircraft noise and the incidence of IHD was investigated in two cohort studies [52,78] and in one case control study [57,76]. As part of the WHO evidence review, no cohort nor case control studies were included that investigated the association between aircraft noise exposure and the incidence of IHD. The WHO evidence review evaluated only one cohort study investigating the association between aircraft noise exposure and mortality due to IHD: the Swiss National Cohort Study (SNC). In this study, a positive but statistically not significant association between air traffic noise and mortality due to IHD was found. Based on the results of this study, an Relative Risk (RR) of 1.04 (95%CI: 0.98–1.11) per 10 dB (L_DEN_) was estimated. In the current overview, the association between aircraft noise and mortality due to IHD was investigated in two studies: new results from the SNC-study (cohort study) [72,73] and the NORAH study (case-control study) [58,76]. The new results of the SNC-study were quite comparable with the findings reported in the WHO evidence review: small increases in risk were often found, which were, depending on adjustment for confounders, borderline significant. In the NORAH study, similar results as in the SNC-study were found.

For road traffic noise, 10 cohort studies [78,79,80,81,82] and one case-control study [58,76] were identified and selected in the current overview, which investigated the association between road traffic noise and the incidence of IHD. One of these cohort studies [78] (the DCH study), was already included in the WHO evidence review, but contained new and/or additional results. As part of the WHO evidence review, in total three cohort studies and four case control studies were included that investigated the association between road traffic noise exposure and the incidence of IHD. 

For the association between road traffic noise and mortality due to IHD, the researchers of the WHO evidence review were able to include one case control study and two cohort studies. After aggregating the results of these studies, an RR of 1.05 (95%CI: 0.97–1.13) per 10 dB (L_den_) increase in road traffic noise was estimated. As part of the new literature search, we found one cohort study and one case control study investigating the association between road traffic noise and mortality due to IHD. The results of none of these newly identified studies were available during the execution of the WHO evidence review.

As part of the WHO evidence review, the researchers were only able to include the results of four cross-sectional studies. For the current overview, only one case-control study [58,76] was identified and selected that investigated the impact of rail traffic noise exposure on both the incidence and mortality of IHD. Additionally, two cohort studies were identified and selected that investigated the impact of rail traffic noise exposure on the risk of IHD: in one of the cohort studies (SNC), the effect on mortality was studied [72,73], while in the other cohort study (CAENS) [77], the association with the incidence of IHD was investigated. 

For wind turbine noise, both the identified and selected cohort studies [84,85] investigated the association between wind turbine noise and the incidence of IHD. During the execution of the WHO evidence review, only the results of three cross-sectional studies were available, investigating the association between exposure to wind turbine noise and self-reported cardiovascular disease.

#### 3.2.3. Studies Investigating the Impact of Environmental Noise on Stroke

Nine studies (eight cohort studies and one case-control study) described in 10 publications [52,57,65,71,72,74,76,77,85,86] looked at the impact of environmental noise on stroke. Within these nine studies, four investigated the impact of aircraft noise [53,58,73,77,86], eight investigated the impact of road traffic noise [53,58,73,77,80,81,82,86], three studied the impact of rail traffic noise [57,67,77,86] and one investigated the impact of wind turbine noise. [71,85] In these nine studies, the incidence or mortality of stroke was ascertained by means of a clinical interview/anamnesis or by means of a health registration database. 

The association between aircraft noise and the incidence of stroke was investigated in two cohorts [53,77] and one case control study [58,86]. As part of the WHO evidence review, only two ecological studies were evaluated, investigating the association between aircraft noise exposure and the incidence of stroke. Furthermore, only one cohort study was included investigating the association between aircraft noise exposure and mortality due to stroke: in the Swiss National Cohort Study (SNC), no association was found between air traffic noise and mortality due to stroke. Based on the results of this study, an RR of 0.99 (95%CI: 0.94–1.04) per 10 dB (L_DEN_) was estimated. In the current overview, we found two studies that investigated the association between aircraft noise and mortality due to stroke: one cohort study presenting updated results from the SNC-study [73] and one case control study (the NORAH study). [58,87] The updated results from the SNC were quite comparable with the results of the SNC study included in the WHO-evidence review; again, small increases in risk were found, which were, depending on adjustments for confounders, borderline significant. In the NORAH study, larger but non-significant increases in risk due to increase in aircraft noise were found.

As part of the WHO evidence review, only one cohort study was included that investigated the association between road traffic noise exposure and the incidence of stroke: in the Danish Diet Cancer and Health Cohort (DCH), a positive and statistically significant association was found between road traffic noise and the incidence of stroke. An RR of 1.14 (95%CI: 1.03–1.25) per 10 dB was estimated. For exposure to road traffic noise, six cohort studies [53,77,80,81,82] and one case-control study [58,86], investigating the association between road traffic noise and the incidence of stroke, were identified and selected for the current overview. 

In the WHO evidence review, three cohort studies were included investigating the association between road traffic noise exposure and mortality due to stroke. After combining the results of these three studies, an RR of 0.87 (95%CI: 0.71–1.06) per 10 dB (L_den_) was estimated. For the current study, two new studies investigating the association between road traffic noise and mortality due to stroke were identified and selected: one cohort study [73] and one case-control study [58,86].

As part of the WHO-evidence review, no cohort nor case control studies were evaluated that investigated the impact of rail traffic noise on the risk of stroke. For the current overview, two studies were identified and selected that investigated the association between rail traffic noise and the incidence of stroke: the NORAH study (case-control study) [57,86] and the CAENS study (cohort study). In the NORAH study, significant increases in the risk of stroke due to rail traffic noise were found; in the CAENS study, no consistent associations were observed between the rail traffic noise exposure and the incidence of stroke. 

In addition, for the current overview, two studies were identified that investigated the association between rail traffic noise and mortality due to stroke: the SNC-study (cohort study) [73] and the NORAH study [58,86]. In the NORAH study, a significant increase in the risk of stroke due to rail traffic noise was found. In the SNC-study no increases in the risk of stroke due to rail traffic noise were found.

Only one study investigated the association between wind turbine noise and the incidence of stroke [71,85]. This cohort was carried out in Denmark, and consisted of a population of 712,402 persons aged between 25–85 years. The researchers did not find convincing evidence for an association between wind turbine exposure and the incidence of stroke. 

#### 3.2.4. Studies Investigating the Impact of Environmental Noise on Diabetes

Compared with other outcomes, there are only three new cohort studies and one case-control study identified and selected that have studied the impact of environmental noise on diabetes [88,89,90,91]. In these four studies the incidence or mortality of diabetes was ascertained as a measurement/clinical interview or healthcare registration. 

Of the four identified and selected studies, two cohort studies investigated the association between aircraft noise and the incidence of diabetes: a follow-up of Greek respondents of the HYENA study and the Swiss cohort study on Air Pollution And Lung and heart Disease (SAPALDIA). As part of the WHO evidence review, only one cohort study was included that investigated the association between air traffic noise exposure and the incidence of diabetes: the Stockholm Diabetes Preventive Program (SDPP). In the SDPP study no association was found: an RR of 0.99 (95%CI: 0.47–2.09) per 10 dB was estimated. The results of the two new cohort studies that investigated the association between aircraft noise exposure and the incidence of diabetes were not consistent. The HYENA study revealed no association between aircraft noise and the incidence of doctor-diagnosed diabetes. However, the results of this study were based on a relatively small number of participants and a small number of incident cases of diabetes. In contrast to the results of the SDPP study and the HYENA study, the researchers of the SAPALDIA study found a positive association between aircraft noise exposure and the incidence of diabetes.

As part of the WHO evidence review, only one cohort study was included that investigated the association between road traffic noise exposure and the incidence of diabetes: the Danish Diet Cancer and Health Cohort (DCH). Based on the data of this cohort an RR of 1.08 (95%CI: 1.02–1.14) per 10 dB was estimated. The current overview revealed three new cohort studies, investigating the association between road traffic noise and diabetes [53,87,88], including new results from the DCH study.

As part of the WHO evidence review, only one cohort study was included that investigated the association between rail traffic noise exposure and the incidence of diabetes: the Danish Diet Cancer and Health Cohort (DCH). Based on the data of this cohort an RR of 0.97 (95%CI: 0.89–1.05) per 10 dB (Lden) was estimated. The current overview revealed two cohort studies, investigating the association between rail traffic noise and the incidence of diabetes [87,88], including new results from the DCH study. Similar to their first results, the researchers of the DCH study again did not find an association between rail traffic noise and the incidence of diabetes. After adjustment for confounders, they found an RR (expressed as HR) of 0.99 (95%CI: 0.94–1.04) per 10 dB for five-year exposure. These results were confirmed by the researchers of the other cohort study that was identified and selected for the current overview: the SAPALDIA study. This study also did not find an association between rail traffic noise and the incidence of diabetes. 

Fur the current review, one study was identified and selected that investigated the association between wind turbine noise during the night and the incidence of diabetes [71,91]. The results of the study do not support an association between night time wind turbine noise and a higher risk of diabetes.

#### 3.2.5. Studies Investigating the Impact of Environmental Noise on (Indicators of) Obesity

Three new cohort studies investigated the association between environmental noise and (indicators of) obesity [92,93,94,95,96]. The indicators of obesity were a change in Body Mass Index, change in waist circumference, weight gain, central obesity, overweight and/or change in percentage body fat.

For the WHO evidence review, only one cohort study was included, which investigated the association between aircraft noise and obesity: the SDPP study. As indicators of obesity, they used changes in Body Mass Index (BMI) and waist circumference. After adjustment for confounders, an increase in BMI of 0.14 (95%CI: −0.18–0.45) kg/m^2^ was found per 10 dB aircraft noise level. Furthermore, they found that an increase of 10 dB in aircraft noise level was associated with an increase in waist circumference of 3.46 (95%CI: 2.13–4.77) cm. In the current overview, we identified and selected two cohort studies investigating the association between aircraft noise and obesity: the SDPP study [94] (presenting new results) and the SAPALDIA [93] study. The new results of the SDPP study confirmed the results of the first analyses; again, an increase in aircraft noise exposure was statistically significant associated with an increase in waist circumference. Instead of a change in BMI, the researchers used other indicators of obesity: weight gain, the incidence of overweight and the incidence of central obesity. All these indicators were statistically significantly associated with aircraft noise. In the SAPALDIA study, although similar indicators of obesity were used as in the SDPP study, a statistically significant association was not confirmed for all indicators. 

The association between road traffic noise and obesity was studied in all three cohort studies [93,94,95]. All these three cohort studies also focused on the association between rail traffic noise and obesity [93,94,95]. Two of the cohort studies (SDPP and DCH) were already included in the WHO evidence review. The WHO review presented the cross-sectional results, whilst the updated results contained longitudinal results.

None of these studies investigated the impact of wind turbine noise on obesity.

#### 3.2.6. Blood Pressure in Children

The new search did not reveal any new studies investigating the association between aircraft noise, rail traffic or wind turbine noise and children’s blood pressure. Only one new study [98] investigated the association between road traffic noise and children’s blood pressure, but this study was of cross-sectional design and was excluded.

## 4. Discussion

The number of relevant studies published after the publication of the WHO reviews on environmental noise, and their effects on annoyance, sleep disturbance, the cardiovascular and metabolic system, was considerable. These include papers identified over the period between January 2014 and June 2019 as well as including some later papers (December 2019) published ahead of print at the time of the search. – In general, the new studies are population based, have a low to medium risk of bias and have a larger geographical spread in comparison to the earlier evidence reviews as far as annoyance and sleep are concerned. In view of quality, for cardiovascular and metabolic outcomes, only case-control and cohort studies were considered for selection. For the other outcomes and sources, the risk for methodological bias was estimated and was generally evaluated as moderate in the studies on environmental noise.

The identified studies include new studies on annoyance and sleep disturbance in relation to environmental noise exposure as well as new results of existing studies. For cardiovascular and/or metabolic outcomes, several new case-control and cohort studies into the association between environmental noise exposure and cardiovascular and/or metabolic outcomes were identified, as well as new publications on studies that were already included in the WHO-evidence review. Below, the findings are summarised in relation to the original WHO reviews and discussed per outcome and their implication for any potential future meta-analysis.

### 4.1. Sleep Disturbance

The systematic review on sleep disturbance [3] covering the period of January 2000–December 2014 identified 74 studies, of which 33 were used in a quantitative meta-analysis. Regarding the strength of the evidence, the authors concluded that the quality of the evidence was “moderate” for an association between road traffic noise and cortical awakenings and self-reported sleep disturbance (for questions that referred to noise). For the other noise sources, the quality was evaluated as “very low” for all investigated sleep outcomes.

Overall, it was concluded that transportation noise affects objectively measured sleep physiology and subjectively assessed sleep disturbance in adults. For other outcome measures and noise sources, the examined evidence was conflicting or only emerging. Research gaps and needs identified are the number, size and generalizability of studies on the effects of noise using objective indicators of sleep. The heterogeneity of the studies limits the value of the generic EErs.The new search revealed 34 studies addressing the effects of environmental noise on sleep, covering the January 2015–December 2019 period. For aircraft noise new evidence from the DEBATS (France) [39,43] and NORAH study (Germany) [20] in relation to sleep disturbance suggest an update. This could also be considered for road and rail traffic noise, although based on the results for these sources, no large differences are to be expected as far as sleep disturbance is concerned. Analogous to the WHO review, these results are in favour of a separate meta-analysis on the objective sleep measures in this case for wind turbine noise. Additionally, the new studies provide more evidence on the role of the number of events and the L_max_ levels and it would be interesting to compare the outcomes from the different new studies including the different noise indicators.

### 4.2. Annoyance

The WHO review identified 62 studies, by means of a systematic review search covering January 2000–December 2013. Of the 62 studies, 46 were used in a quantitative meta-analysis. Regarding the quality of the evidence, the associations between aircraft noise levels and percentage highly annoyance (%HA) and the associations between road traffic noise and %HA were graded as “moderate” [4]. The quality of evidence for an association between rail traffic noise and percentage %HA was judged as “moderate” to “high”, while the association between wind turbines noise and %HA was “low”. Several research gaps were identified, such as the variance in the characterisation of exposure and the measurement and ascertainment of %HA as the main sources of heterogeneity. Additionally, only very few studies on wind turbines were available during that time period.

Our new search revealed 39 (sub) studies with “moderate” to “high” quality, studying the effects of environmental noise on annoyance covering the period of 2015–2019. Results warrant a new update of the meta-analysis on annoyance for aircraft noise, road traffic, rail traffic noise and wind turbine noise. For aircraft noise, new evidence from the DEBATS (France) [39,42] and NORAH study (Germany) [20] in relation to annoyance suggests an update of the WHO guidelines. This could also be considered for road and rail traffic noise, although for these sources, no large differences are to be expected as far as annoyance reactions are concerned.

### 4.3. Cardiovascular Outcomes

For the evidence review on cardiovascular effects [6,7] the literature search covered the period of January 2000–December 2014. In total, 61 studies were identified and 53 of these were used in the quantitative meta-analyses. Regarding the quality of the studies, it was concluded that the majority of the studies concerned road traffic noise and hypertension, with a cross-sectional design. Despite the fact that most of these studies adjusted for important confounders, and were capable to ascertain individual exposure levels, the quality of the evidence was rated as “very low”. The most comprehensive evidence was available for road traffic noise and Ischemic Heart Diseases (IHD), which revealed a significant association. The quality of the evidence based on these longitudinal studies was rated as “high”. A research gap identified was that for a comprehensive assessment of the impact of noise exposure on the cardiovascular system, more and of better quality evidence is needed. This can best be provided by case-control and cohort studies.

The new search revealed 30 new (sub-) studies into the effect of environmental noise on the cardiovascular system covering the period between 2015 and mid-2019. Results suggest an update of the meta-analysis for the association between aircraft noise and hypertension (incidence), IHD (incidence) and stroke (incidence), hereby applying the rule of thumb of at least three studies. For road traffic noise the new studies indicate the potential of an update of the meta-analysis for the association between road traffic noise and hypertension (incidence), IHD (incidence), IHD (mortality), stroke (incidence) and stroke (mortality). The results also warrant an update of the meta-analyses on hypertension (incidence) for rail traffic noise. Although it is unlikely that the new results will change the conclusions of the WHO evidence review with regard to the association between rail traffic noise and the incidence of hypertension, a systematic evaluation and meta-analysis can be applied to demonstrate this hypothesis. The number of high-quality studies on the effect of wind turbine noise is too limited to justify a meta-analysis. 

Overall, a systematic evaluation and meta-analysis will clarify whether and how the results of the newly found studies affect the conclusions of the WHO review with regard to aircraft, road traffic and rail traffic noise and IHD. For the association between wind turbine and IHD, there is still not enough evidence to justify a meta-analysis on these data.

A systematic evaluation and meta-analysis will confirm whether and how the results of the newly found studies affect the conclusions of the WHO review with regards to aircraft noise and road traffic noise on stroke. A systematic evaluation and meta-analysis would demonstrate whether and how the results of these newly found studies affect the conclusions of the WHO review with regard to road traffic noise and the incidence of hypertension. Given the number of eligible studies, it is not recommended to carry out a meta-analysis for the effect of rail traffic noise and wind turbine noise on the incidence or mortality of stroke.

### 4.4. Metabolic Outcomes

Eight studies were used in the quantitative meta-analyses after a systematic review search covering the period of January 2000–December 2014 [6,7]. Only a few studies reported on the association between transportation noise exposure and diabetes and/or obesity. The authors rated the quality of evidence for an association from “moderate” to “very low”, depending on noise source and outcome, primarily based on longitudinal studies. It was also concluded that for a comprehensive assessment of the impact of noise exposure on the metabolic system, more and of better quality evidence is needed.

The new search revealed eight new studies into the effect of noise exposure on the metabolic system, covering the period between January 2015 and mid–2019. Based on these new studies, an update of some meta-analyses is suggested. These include a meta-analysis for the association between aircraft noise exposure and diabetes (incidence) and road traffic noise exposure and diabetes (incidence), as well as changes in BMI and changes in WC. The number of studies is too limited to justify a new meta-analysis on the association between aircraft and road traffic noise exposure and mortality. For the association between aircraft and road traffic noise exposure and the incidence of diabetes, three studies were available in total, which makes it worthwhile to carry out a meta-analysis. For rail traffic noise and wind turbine noise, the number of studies is too limited to justify a new meta-analysis. For the association between road traffic noise exposure and the change in body mass index, three studies were available in total, which makes it worthwhile to carry out a meta-analysis.

### 4.5. Strength and Limitations

Although the grading system was not applied due to time and material limits, the strengths of this synthesis is the rigorous search and selection strategy used. This led to the identification of a large number of recent studies into the health effects of transport related noise sources and noise of wind turbines.

Within the limited time available for this scoping synthesis, it was a challenge to screen the many full papers. We had to compromise sometimes at the cost of the level of detail. Some cardiovascular and metabolic effects, such a screening, had been partly performed earlier, which shows in the level of detail in some of the presented materials within this domain as compared to the other parts of the review (Supplementary 2, Tables S2.1–S2.18). On the other hand, data extraction was more extensive for annoyance and sleep disturbance. The present scoping synthesis focused on studies that investigated sleep disturbance, annoyance, cardiovascular and metabolic effects as primary outcomes, and objectively measured or estimated noise levels as a primary predictor. However, several studies were identified that considered noise exposure or annoyance as a confounder or mediator/moderator, and therefore, effect sizes of noise-outcome associations were not provided in the published article. These studies were excluded in the current review update; however, it might be worthwhile to reconsider them in future meta-analyses, under the condition that the necessary data are available.

Risk of bias assessment was estimated for the studies dealing with annoyance and sleep disturbance. It primarily focused on aspects such as exposure misclassification, selective participation and confounding as proposed by Grimes and Schulz [12]. The rating method was qualitative and comparable to schemes applied in recent systematic evaluations of the observational literature on different exposures [13,99,100]. However, this assessment was not a prerequisite for the consideration of a study as eligible for inclusion. Besides the risk of bias, at a later stage, further and more elaborate evaluation of study quality, based on a validated instrument specialised in observational research, is needed.

### 4.6. Implication for Future Research

To evaluate whether an update of the reviews underlying the WHO Environmental Noise guidelines and derived Exposure Response relations is needed and/or should be extended, criteria were formulated to make a statement about whether the use or need for adaptation of the Exposure Response relationships and/or risk ratios should be considered by the IGCB(N) for cardiovascular, metabolic effects, annoyance and sleep. Such an update should include previous evidence from the WHO reviews and the meta analyses to be performed based on the total evidence.

To make a statement suggesting whether new meta-analyses aimed at confirming or adapting existing EErs is justified, we used our professional judgement. Study size, response rates, design, quality/risk of bias, and the way in which the exposure and outcome were measured or estimated, and the confounders considered all played a role in this evaluation. For cardiovascular and metabolic outcomes in addition only case control and cohort studies (= high quality design) were considered. This restriction was not applied to sleep and annoyance. Additionally, the rule of thumb was followed that a meta-analysis is only advised when at least three studies are available.

This review calls for a closer look at the broad geographic spread, the number, size and quality of the new studies before deciding whether new meta-analyses are needed. After this, when it has been decided to perform new meta-analyses, several issues need to be considered. In view of studying heterogeneity and to perform the meta-analyses properly, it is crucial to obtain accurate data with comparable cut-off points in outcomes where relevant (e.g., % HA, HSD) in cases where these are not derivable from the publications. Communication with the original authors often constitutes a great challenge and a time-consuming process. 

## 5. Conclusions

This scoping synthesis was performed to draw conclusions about the need for an update of the exposure-outcome associations derived in the noise reviews published in the context of the WHO Guidelines [2]. Results showed that since 2014, an impressive number of articles was published addressing the association between transport related noise exposure and wind turbine noise exposure and annoyance, sleep disturbance and metabolic and cardiovascular effects. The average quality is “moderate” to “high” (with regard to sleep disturbance and annoyance), and remarkable is the broad geographic spread of the new studies. The number and size of the new studies warrant new meta-analyses, in particular where the cardiovascular effects are concerned, but also for annoyance and sleep disturbance. For the cardiovascular and metabolic effects, the recent meta-analysis by Vienneau et al. [100] should hereby be taken into account. Differences in effect due to the in- or exclusion of different types of study should also be discussed in more detail in view of the current discussion [101,102,103]. In addition, the new evidence regarding wind turbine noise exposure and effects would justify meta-analyses on annoyance and sleep related effects. Overall, it is worthwhile to have a closer look at the transport related source-specific new findings on annoyance and sleep disturbance before deciding whether new meta-analyses are needed.

## Figures and Tables

**Table 1:** Search profile.

1	Published or Accepted Papers in Peer-Review Journals
**2**	Published papers in conference proceedings
**3**	Individual studies, thus., no reviews, meta-analyses or “commentaries”
**4**	Language: Dutch, German, English and French
**5**	Population: general population, adults; (cardiovascular effects also include children, for other outcomes not relevant or available)
**6**	Setting: Environmental exposure at home or at school (for children) only (NO exposure to noise in occupational setting nor in health care setting (e.g., hospital))
**7**	Study design: observational studies only (NO experimental studies following the WHO protocol), For the update on Cardiovascular effects and metabolic effects only case control studies and cohort studies are selected
**8**	Relevant outcomes: annoyance, sleep disturbance, (self-reported or clinically diagnosed) blood pressure, hypertension, coronary heart disease, stroke, diabetes and indicators of obesity

## Supplementary Materials

The following are available online at https://www.mdpi.com/1660-4601/17/9/3016/s1, Supplementary 1: Search profiles Part 1: Annoyance and Sleep disturbance -Road, Rail, Air, Wind turbines (update since 2014); Part 2a Air/rail noise and hypertension/cardiovascular diseases (Update search 2014); Part 2b Aircraft and/or rail traffic and/or road traffic noise and stroke/diabetes/obesity (Update search 2014); Part2c: Road traffic noise and blood pressure/hypertension (update search 2014); Part 2d: Traffic noise and blood pressure in children (Update search 2014); Part 2e: Noise wind turbines and blood pressure/cardiovascular diseases (Update search 2014). Supplementary 2: Tables: Segment 1. Table S1.1 Overview of the characteristics of the selected studies on the association between road traffic noise and annoyance; Table S1.2 Overview of the characteristics of the selected studies on the association between air traffic noise and annoyance. Table S1.3 Overview of the characteristics of the selected studies on the association between rail traffic noise and annoyance. Table S1.4 Overview of the characteristics of the selected studies on the association between wind turbine noise and annoyance. Table S1.5 Overview of the characteristics of the selected studies on the association between combined sources and annoyance. Table S1.6 Overview of the characteristics of the selected studies on the association between road traffic noise and sleep disturbance. Table S1.7 Overview of the characteristics of the selected studies on the association between air traffic noise and sleep disturbance. Table S1.8 Overview of the characteristics of the selected studies on the association between wind turbine noise and sleep disturbance. Table S1.9 Overview of the characteristics of the selected studies on the association between mixed noise sources and annoyance, sleep disturbance. Segment 2. Table S2.1 Overview of the characteristics of the selected studies on the association between aircraft noise and hypertension. Table S2.2 Overview of the characteristics of the selected studies on the association between road traffic noise and hypertension. Table S2.3 Overview of the characteristics of the selected studies on the association between rail traffic noise and hypertension. Table S2.4 Overview of the characteristics of the selected studies on the association between wind turbine noise and hypertension. Table S2.5 Overview of the characteristics of the selected studies on the association between aircraft noise and ischemic heart disease. Table S2.6 Overview of the characteristics of the selected studies on the association between road traffic noise and ischemic heart disease. Table S2.7 Overview of the characteristics of the selected studies on the association between rail traffic noise and ischemic heart disease. Table S2.8 Overview of the characteristics of the selected studies on the association between wind turbine noise and ischemic heart disease. Table S2.9 Overview of the characteristics of the selected studies on the association between air traffic noise and stroke. Table S2.10 Overview of the characteristics of the selected studies on the association between road traffic noise and stroke. Table S2.11 Overview of the characteristics of the selected studies on the association between rail traffic noise and stroke. Table S2.12 Overview of the characteristics of the selected studies on the association between aircraft noise and diabetes. Table S2.13 Overview of the characteristics of the selected studies on the association between road traffic noise and diabetes. Table S2.14 Overview of the characteristics of the selected studies on the association between rail traffic noise and diabetes. Table S2.15 Overview of the characteristics of the selected studies on the association between wind turbine noise and diabetes. Table S2.16 Overview of the characteristics of the selected studies on the association between aircraft noise and indicators of obesity. Table S2.17 Overview of the characteristics of the selected studies on the association between road traffic noise and indicators of obesity. Table S2.18 Overview of the characteristics of the selected studies on the association between rail traffic noise and obesity. Supplementary 3: Glossary.
