# Supplementary material for: Evidence Relating to Environmental Noise Exposure and Annoyance, Sleep Disturbance, Cardio-Vascular and Metabolic Health Outcomes in the Context of IGCB (N): A Scoping Review of New Evidence
Source: Int J Environ Res Public Health. 2020 Apr 26;17(9):3016. doi: 10.3390/ijerph17093016 (PMC7246943; doi:10.3390/ijerph17093016)
Supplement: Supplementary file 1 [file ijerph-17-03016-s001.pdf]

Supplementary 1: Search profiles

Period: 26/03/2019-24/05/2019 by Jeanine Ridder (RIVM)

Part 1: Annoyance and Sleep disturbance -Road, Rail, Air, Windturbines (update since 2014)

Embase

Query	Results	No.
#18	#17 AND [2014-2019]/py	240
#17	#12 AND #16	586
#16	#13 OR #14 OR #15	264,858
#15	'annoyance':ti OR 'sleep*':ti	128,985
#14	'annoyance'/exp	1,233
#13	'sleep'/exp OR 'sleep disorder'/exp	341,988
#12	#6 OR #11	5,318
#11	(#7 OR #8 OR #9) AND (#7 OR #10)	177
#10[1]	'noise'/exp OR 'sound'/exp OR 'noise pollution'/de OR 'infrasound'/exp OR infrasound*:ti,ab OR noise*:ti,ab OR 'low frequen*':ti,ab	399,689
#9	'wind'/exp AND ('renewable energy'/de OR 'electric power plant'/de OR 'power supply'/exp OR 'energy resource'/de)	308
#8	'wind power'/exp OR 'wind farm'/exp	655
#7	noise AND ((wind NEAR/3 turbine*) OR (wind NEAR/3 farm*) OR windturbine* OR windfarm* OR 'wind park*' OR 'wind mill*' OR windpark* OR windmill*)	182
#6	(#1 OR #2 OR #3 OR #5) AND (#1 OR #4 OR #5)	5,160
#5	'traffic noise'/exp OR 'aircraft noise'/exp	2,505
#4	'noise'/mj OR 'sound'/mj OR 'vibration'/mj	32,711
#3	'aircraft'/exp OR 'airport'/exp OR 'railway'/exp OR 'motor vehicle'/exp	54,095
#2	'traffic and transport'/exp/mj	100,313
#1	noise NEAR/5 (rail* OR aircraft OR airport* OR road* OR traffic* OR automobile* OR vehicle* OR motorcycle* OR transport*)	4,712

Ti: in title/Mj Major , main topic of paper /Exp not all keywords have to be mentioned separately

[1] The search for DEFRA assignment also included neighbour, neighbourhood, industrial, and low frequency noise Marked in shaded colour

Scopus

((TITLE-ABS-KEY (noise W/5 (rail* OR aircraft OR airport* OR road* OR traffic* OR automobile* OR vehicle* OR motorcycle*))) AND (TITLE (sleep* OR annoyance) OR KEY (noise-annoyance)) AND PUBYEAR > 2013) OR ((TITLE-ABS-KEY (noise) AND TITLE-ABS-KEY ((wind W/3 turbine*) OR windturbine* OR (wind W/3 farm*) OR windfarm* OR wind-park* OR windpark* OR wind-mill*)) AND (TITLE (sleep* OR annoyance) OR KEY (sleep* OR annoyance)) AND PUBYEAR > 2013)

PubMed

Search	Query	Items found
#21	Search **#20 AND 2014:2019[dp]**	177
#20	Search **#17 AND (#18 OR #19)**	514
#19	Search **annoyance[ti] OR sleep*[ti]**	88617
#18	Search **"Sleep"[mh] OR "Sleep Wake Disorders"[mh]**	131779
#17	Search **#6 OR #16**	5911
#16	Search **(#7 OR #13) AND (#7 OR #14 OR #15)**	175
#15	Search **infrasound*[tiab] OR noise[tiab] OR "low frequen*"[tiab]**	127874
#14	Search **"Noise"[mh] OR "Sound"[mh]**	38465
#13	Search **#8 AND (#9 OR #10 OR #11 OR #12)**	345
#12	Search **"Energy-Generating Resources"[mh:noexp]**	2077
#11	Search **"Electric Power Supplies"[mh:noexp]**	6837
#10	Search **"Power Plants"[mh:noexp]**	6029
#9	Search **"Renewable energy"[mh:noexp]**	745
#8	Search **"Wind"[mh]**	4139
#7	Search **noise[tiab[AND ("wind turbine*"[tiab] OR "windturbine*"[tiab] OR "wind farm*"[tiab] OR "windfarm*"[tiab] OR "wind park*"[tiab] OR windpark*[tiab] OR "wind mill*"[tiab] OR windmill*[tiab])**	141
#6	Search **(#1 OR #2 OR #3 OR #5) AND (#1 OR #4 OR #5)**	5764
#5	Search **"Noise,transportation"[mh]**	1326
#4	Search **"Noise"[mj:noexp]**	11924
#3	Search **"Aircraft"[mh:noexp] OR "Airports"[mh:noexp] OR "Railroads"[mh:noexp] OR "Motor vehicles"[mh]**	30480
#2	Search **"Transportation"[majr]**	44470

Search	Query	Items found
#1	Search **noise[tiab] AND (rail*[tiab] or aircraft[tiab] or airport*[tiab] or road*[tiab] or traffic*[tiab] or automobile*[tiab] or vehicle*[tiab] or "motor cycle*"[tiab] or motorcycle*[tiab] or transport*[tiab])**	5046

PsycINFO

#	Searches	Results
1	(noise adj5 (rail* or aircraft or airport* or road* or traffic* or automobile* or vehicle* or motorcycle*)).mp. [mp=title, abstract, heading word, table of contents, key concepts, original title, tests & measures]	445
2	traffic.mp. or aircraft/ or railroad trains/ or transportation/ or motor vehicles/	18859
3	exp Noise Effects/	3294
4	exp Auditory Stimulation/	28344
5	exp VIBRATION/	1450
6	(noise*or infrasound or low frequen*).ti,ab.	9729
7	((wind adj3 turbine*) or windturbine* or (wind adj3 farm*) or windfarm* or (wind adj3 park*) or windpark* or windmill* or wind mill*).mp. [mp=title, abstract, heading word, table of contents, key concepts, original title, tests & measures]	194
8	(1 or 2) and (1 or 3 or 4 or 5 or 6)	637
9	7 and (3 or 4 or 5 or 6)	12
10	8 or 9	647
11	exp Sleep Disorders/ or exp Sleep/ or exp Sleepiness/ or exp Sleep Deprivation/	34770
12	(annoyance or sleep*).mp. [mp=title, abstract, heading word, table of contents, key concepts, original title, tests & measures]	77655
13	11 or 12	79012
14	10 and 13	145
15	limit 14 to yr="2014-current"	21

Part 2a: Air/rail noise and hypertension/cardiovascular diseases (Update search 2014) - 26032019

Embase

Query	Results	No.
#19	#18 AND [2014-2019]/py	109
#18	#17 NOT child*:ti	263
#17	#15 AND #16	268
#16	#8 OR #9 OR #10 OR #11 OR #12	4,488,433
#15	#13 AND #14	1,958
#14	#1 OR #2 OR #6 OR #7	18,670
#13	#1 OR #2 OR #3 OR (#4 AND #5)	16,593
#12	'ischaemic heart disease*' OR 'ischemic heart disease*' OR 'coronary heart disease*' OR 'angina pectoris' OR 'myocard* infarct*' OR 'cardiovascular disease*' OR 'heart disease*'	1,041,266
#11	'hypertension' OR 'blood-pressure'	1,233,280
#10	'cardiovascular disease'/exp	4,098,866
#9	'hypertension'/exp	695,400
#8	'blood pressure'/exp	549,391
#7	'traffic noise'/exp	2,019
#6	'noise'/mj	16,113
#5	rail* OR 'aircraft' OR airport* OR 'air traffic'	39,708
#4	'traffic and transport'/mj	3,741
#3	'aircraft'/exp OR 'airport'/exp OR 'railway'/exp	15,746
#2	'aircraft noise'/exp	616
#1	noise NEAR/5 (rail* OR aircraft OR airport* OR 'air traffic*')	1,652

Scopus

((TITLE-ABS-KEY ((rail* OR aircraft OR airport* OR air-traffic*) W/5 noise)) AND (TITLE-ABS-KEY (hypertension OR blood-pressure OR ischemic-heart-disease* OR coronary-heart-disease* OR angina-pectoris OR myocard*-infarct* OR cardiovascular-disease* OR heart-disease*)) AND PUBYEAR > 2013) AND NOT (TITLE (child*))

PubMed

Search	Query	Items found
#16	Search #15 AND 2014:2019[dp]	61
#15	Search #14 NOT "child*"[ti]	186
#14	Search #13 AND (#7 OR #8 OR #9 OR #10 OR #11)	186
#13	Search #12 AND (#1 OR #5 OR #6)	1425

Search	Query	Items found
#12	Search (#1 OR #2 OR (#3 AND #4)	12269
#11	Search "ischaemic heart disease*"[tiab] or "ischemic heart disease"[tiab] or "coronary heart disease*"[tiab] or "angina pectoris"[tiab] or "myocard* infarct*"[tiab] or "cardiovascular disease*"[tiab] or "heart disease*"[tiab]	276684
#10	Search "hypertension"[tiab] or "blood pressure"[tiab]	557460
#9	Search "Cardiovascular diseases"[mh]	2258495
#8	Search "Hypertension"[mh]	244168
#7	Search "Blood pressure"[mh]	280805
#6	Search "Noise,transportation"[mh:noexp]	1320
#5	Search "Noise"[mj:noexp]	11899
#4	Search rail*[tiab] or aircraft[tiab] or airport*[tiab] or "air traffic"[tiab]	15275
#3	Search "Transportation"[mj:noexp]	4932
#2	Search "Aircraft"[mh:noexp] OR "Airports"[mh:noexp] OR "Railroads"[mh:noexp]	11604
#1	Search noise[tiab] AND (rail*[tiab] or aircraft[tiab] or airport*[tiab] or "air traffic"[tiab])	1121

Part 2b Aircraft and/or rail traffic and/or road traffic noise and stroke/diabetes/obesity (Update search 2014)

Embase

Query	Results	No.
#15	#14 AND [2014-2019]/py	131
#14	#13 NOT child*:ti	196
#13	#6 AND #12	205
#12	#7 OR #8 OR #9 OR #10 OR #11	2,426,661
#11	'diabetes' OR 'obesit*' OR 'overweight' OR 'bmi' OR 'body mass index'	1,611,441
#10	'stroke' OR 'cerebrovascular accident*' OR 'cva' OR 'cerebrovascular disorder*' OR 'brain vascular accident*' OR 'brain vascular disorder*'	500,485
#9	'obesity'/exp OR 'body mass'/exp	713,875
#8	'diabetes mellitus'/exp	908,691
#7	'cerebrovascular disease'/exp	670,481
#6	(#1 OR #2 OR #3) AND (#1 OR #4 OR #5)	4,671
#5	'traffic noise'/exp OR 'aircraft noise'/exp	2,504
#4	'noise'/mj	16,113
#3	'aircraft'/exp OR 'airport'/exp OR 'railway'/exp OR 'motor vehicle'/exp	54,074
#2	'traffic and transport'/exp/mj	100,284
#1	noise NEAR/5 (rail* OR aircraft OR airport* OR traffic* OR automobile* OR vehicle*)	4,175

Scopus 20190326

((TITLE-ABS-KEY ((rail* OR aircraft OR airport* OR road* OR traffic* OR automobile* OR vehicle*) W/1 noise)) AND (TITLE-ABS-KEY (stroke OR cerebrovascular OR cva OR brain-vascular OR diabetes OR obesit* OR overweight OR bmi OR body-mass-index)) AND PUBYEAR > 2013) AND NOT (TITLE (child*))

PubMed 20190326

Search	Query	Items found
#15	Search #**14 AND 2014:2019[dp]**	88
#14	Search #**13 NOT "child*"[ti]**	151
#13	Search #**6 AND #12**	151
#12	Search #**7 OR #8 OR #9 OR #10 OR #11**	1393382
#11	Search **diabetes[tiab] or obesit*[tiab] or overweight[tiab] or bmi[tiab] or "body mass index"[tiab]**	786753
#10	Search **stroke[tiab] or cerebrovascular*[tiab] or cva[tiab] or "brain vascular accident*"[tiab] or "brain vascular disorder*"[tiab]**	260145

Search	Query	Items found
#9	Search **"Obesity"[mh] or "Overweight"[mh] or "Body Mass Index"[mh]**	269289
#8	Search **"Diabetes Mellitus"[mh]**	398690
#7	Search **"Cerebrovascular disorders"[mh]**	343933
#6	Search **(#1 or #2 or #3) AND (#1 or #4 or #5)**	3663
#5	Search **"Noise,transportation"[mh]**	1320
#4	Search **"Noise"[mj:noexp]**	11899
#3	Search **"Aircraft"[mh:noexp] OR "Airports"[mh:noexp] OR "Railroads"[mh:noexp] OR "Motor vehicles"[mh:noexp]**	15968
#2	Search **"Transportation"[majr]**	44421
#1	Search **noise[tiab] AND (rail*[tiab] or aircraft[tiab] or airport*[tiab] or road*[tiab] or traffic*[tiab] or automobile*[tiab] or vehicle*[tiab])**	3119

Part2c: Road traffic noise and blood pressure/hypertension (update search 2014)

Embase

Query	Results	No.
#12	#11 AND [2014-2019]/py	99
#11	#10 NOT child*:ti	260
#10	#6 AND (#7 OR #8 OR #9)	274
#9	'hypertension' OR 'blood-pressure'	1,233,687
#8	'hypertension'/exp	695,655
#7	'blood pressure'/exp	549,548
#6	(#1 OR #2 OR #3) AND (#1 OR #4 OR #5)	4,353
#5	'traffic noise'/exp	2,021
#4	'noise'/mj	16,113
#3	'motor vehicle'/exp	39,073
#2	'traffic and transport'/exp/mj	100,284
#1	noise NEAR/5 (road* OR traffic* OR automobile* OR vehicle* OR 'motor cycle*' OR motorcycle* OR transport*)	3,667

Scopus

(TITLE-ABS-KEY ((road* OR traffic* OR automobile* OR vehicle* OR motor-cycle* OR motorcycle* OR transport*) W/1 noise)) AND (TITLE-ABS-KEY (hypertension OR blood-pressure)) AND PUBYEAR > 2013 AND NOT TITLE (child*)

PubMed

Search	Query	Items found
#12	Search **#11 AND 2014:2019[dp]**	84
#11	Search **#10 NOT "child*"[ti]**	236
#10	Search **#6 AND (#7 OR #8 OR #9)**	236
#9	Search **"hypertension"[tiab] or "blood pressure"[tiab]**	557533
#8	Search **"Hypertension"[mh]**	244174
#7	Search **"Blood pressure"[mh]**	280818
#6	Search **(#1 or #2 or #3) AND (#1 or #4 or #5)**	5153
#5	Search **"Noise,transportation"[mh]**	1320
#4	Search **"Noise"[mj:noexp]**	11903
#3	Search **"Motor vehicles"[mh]**	19528
#2	Search **"Transportation"[majr]**	44425
#1	Search **noise[tiab] AND (road*[tiab] or traffic*[tiab] or automobile*[tiab] or vehicle*[tiab] or "motor cycle*"[tiab] or motorcycle*[tiab] or transport*[tiab])**	4321

Part 2d: Traffic noise and blood pressure in children (Update search 2014)

Embase

Query	Results	No.
#14	#10 AND #13	52
#13	#11 OR #12	4,007,781
#12	child*:ti,ab OR infant*:ti,ab OR adolescent*:ti,ab	2,152,259
#11	'child'/exp OR 'adolescent'/exp	3,471,333
#10	#6 AND (#7 OR #8 OR #9)	325
#9	'hypertension' OR 'blood-pressure'	1,234,012
#8	'hypertension'/exp	695,863
#7	'blood pressure'/exp	549,677
#6	(#1 OR #2 OR #3) AND (#1 OR #4 OR #5)	4,760
#5	'traffic noise'/exp OR 'aircraft noise'/exp	2,504
#4	'noise'/mj	16,113
#3	'aircraft'/exp OR 'airport'/exp OR 'railway'/exp OR 'motor vehicle'/exp	54,074
#2	'traffic and transport'/exp/mj	100,284
#1	noise NEAR/5 (rail* OR aircraft OR airport* OR road* OR traffic* OR automobile* OR vehicle*)	4,272

Scopus

TITLE-ABS-KEY ((rail* OR aircraft OR airport* OR road* OR traffic* OR automobile* OR vehicle*) W/1 noise) AND TITLE-ABS-KEY (blood-pressure OR hypertension) AND TITLE-ABS-KEY (child* OR infant* OR adolescent*) AND PUBYEAR > 2013

PubMed

Search	Query	Items found
#14	Search **#11 AND 2014:2019[dp]**	17
#12	Search **#10 AND (child OR children OR infant OR infants OR adolescent OR adolescents)**	59
#11	Search **#10 AND (child* OR infant* OR adolescent*)**	61
#10	Search **#6 AND (#7 OR #8 OR #9)**	235
	Search **"hypertension"[tiab] or "blood pressure"[tiab]**	557615
#8	Search **"Hypertension"[mh]**	244185
#7	Search **"Blood pressure"[mh]**	280825
#6	Search **(#1 or #2 or #3) AND (#1 or #4 or #5)**	3663
#5	Search **"Noise,transportation"[mh]**	1322
#4	Search **"Noise"[mj:noexp]**	11904
#3	Search **"Aircraft"[mh:noexp] OR "Airports"[mh:noexp] OR "Railroads"[mh:noexp] OR "Motor vehicles"[mh:noexp]**	15972
#2	Search **"Transportation"[majr]**	44427
#1	Search **noise[tiab] AND (rail*[tiab] or aircraft[tiab] or airport*[tiab] or road*[tiab] or traffic*[tiab] or automobile*[tiab] or vehicle*[tiab])**	3119

Part 2e: Noise wind turbines and blood pressure/cardiovascular diseases *(Update search 2014)*

Embase

Query	Results	No.
#16	#15 AND [2014-2019]/py	30
#15	#8 AND (#9 OR #10) AND (#11 OR #12 OR #13 OR #14)	43
#14	health*:ti	815,872
#13	'cardiovascular function'/exp OR 'cardiovascular disease'/exp OR 'cardiovascular system'/exp	5,316,590
#12	'blood pressure'/exp	549,677
#11	'blood pressure' OR cardiovascular*	1,806,850
#10	infrasound* OR noise* OR 'low frequenc*'	220,783

#9	'noise'/exp OR 'sound'/exp	283,427
#8	#1 OR #2 OR #7	1,131
#7	#3 AND (#4 OR #5 OR #6)	308
#6	'power supply'/exp OR 'energy resource'/de	21,068
#5	'electric power plant'/de	7,827
#4	'renewable energy'/de	2,505
#3	'wind'/exp	8,380
#2	'wind power'/exp OR 'wind farm'/exp	655
#1	(wind NEAR/3 turbine) OR (wind NEAR/3 farm) OR windturbine* OR windfarm*	467

Scopus

TITLE-ABS-KEY ((wind W/3 turbine*) OR windturbine* OR (wind W/3 farm*) OR windfarm*) AND TITLE-ABS-KEY (noise OR infrasound* OR low-frequenc*) AND (TITLE-ABS-KEY (blood-pressure OR cardiovascular*) OR TITLE (health*)) AND PUBYEAR > 2013

PubMed

Search	Query	Items found
#16	Search **#15 AND 2014:2019[dp]**	37
#15	Search **#8 AND (#9 OR #10) AND (#11 OR #12 OR #13 OR #14)**	48
#14	Search **"health"[ti]**	564308
#13	Search **"Cardiovascular Physiological Phenomena"[mh] OR "Cardiovascular Diseases"[mh] OR "Cardiovascular System"[mh]**	3185477
#12	Search **"Blood pressure"[mh]**	280825
#11	Search **"blood pressure"[tiab] OR "cardiovascular*"[tiab]**	629288
#10	Search **infrasound*[tiab] OR noise[tiab] OR "low frequenc*"[tiab]**	127595
#9	Search **"Noise"[mh] OR "Sound"[mh]**	38411
#8	Search **#1 OR #7**	679
#7	Search **#2 AND (#3 OR #4 OR #5 OR #6)**	344
#6	Search **"Energy-Generating Resources"[mh:noexp]**	2076
#5	Search **"Electric Power Supplies"[mh:noexp]**	6831
#4	Search **"Power Plants"[mh:noexp]**	6024
#3	Search **"Renewable energy"[mh:noexp]**	742
#2	Search **"Wind"[mh]**	4127

Search	Query	Items found
#1	Search **"wind turbine*"[tiab] OR "wind farm*"[tiab] OR "windturbine*"[tiab] OR "windfarm*"[tiab]**	473

PsycINFO <1806 to April Week 1 2019>

#	**Searches**	**Results**
1	(noise adj5 (rail* or aircraft or airport* or road* or traffic* or automobile* or vehicle* or motorcycle*)).mp. [mp=title, abstract, heading word, table of contents, key concepts, original title, tests & measures]	445
2	traffic.mp. or aircraft/ or railroad trains/ or transportation/ or motor vehicles/	18859
3	exp Noise Effects/	3294
4	exp Auditory Stimulation/	28344
5	exp VIBRATION/	1450
6	(noise*or infrasound or low frequen*).ti,ab.	9729
7	((wind adj3 turbine*) or windturbine* or (wind adj3 farm*) or windfarm* or (wind adj3 park*) or windpark* or windmill* or wind mill*).mp. [mp=title, abstract, heading word, table of contents, key concepts, original title, tests & measures]	194
8	(1 or 2) and (1 or 3 or 4 or 5 or 6)	637
9	7 and (3 or 4 or 5 or 6)	12
10	8 or 9	647
11	exp Sleep Disorders/ or exp Sleep/ or exp Sleepiness/ or exp Sleep Deprivation/	34770
12	(annoyance or sleep*).mp. [mp=title, abstract, heading word, table of contents, key concepts, original title, tests & measures]	77655
13	11 or 12	79012
14	10 and 13	145
15	limit 14 to yr="2014-current"	21

Supplementary 2: Tables

Segment 1

Table S1. 1 Overview of the characteristics of the selected studies on the association between road traffic noise and annoyance.

9	Country*	Design†	Sample size (response rate%)	Exposure type and assessment	Outcome type and assessment	Confounders considered in analyses	Findings	Bias Risk*
**Banerjee, D. et al., 2013** [14]	Ind	CS	221<br>Response rate unknown	Road traffic Measurements in 5 dB categories between 60-80 dBA Lden	Annoyance	Age , length of residence	Significant for women≥65 dB (A) 45.4 (% HA) 2.73 (95% CI 1.89-6.26) 2.35 (95% CI 0.99-5.58)<br>But not in men : ≥65 dB (A) 50.0 (%HA)<br>1.61 (95% CI 0.75-3.47) 1.41 (95% CI 0.57-3.50)	Medium
**Bunnakrid, K. et al., 2017**[15]	Tha	CS	253<br>Response rate unknown	Road traffic Noise measures at specific point<br>3 times for 24 hours + traffic	Annoyance	Age, length of residence	Mean scores at area level noise levels (per area) not very precise	Medium

9	Country*	Design†	Sample size (response rate%)	Exposure type and assessment	Outcome type and assessment	Confounders considered in analyses	Findings	Bias Risk*
				volumes counted			and very large variation between areas, indication imprecision of the noise estimates	
**Camusso, C. et al., 2016** [16]	Ita	CS	830 Response rate unknown	Road traffic (measured)	Annoyance	Urban Morphology, with and without trams, composition of traffic etc. broad versus narrow streets , attitudes	Noise levels and annoyance show a weak correlation (rho= max .30) Social class important mediator	Medium
**Ragettli, M.S et al., 2015** [17]	Ca	CS	4336 (47)	Road, and total traffic A-weighted outdoor noise levels (LAeq24h) and day-evening-	Annoyance	Age, gender, education, distance to the source	Prevalence: Proportion Ratios (PPR) for highly disturbed people of 1.10 (95% CI: 1.07–1.13) per 1 dB Lden	Low

Table S1. 2 Overview of the characteristics of the selected studies on the association between air traffic noise and annoyance.

Pub.	Country*	Design†	Sample size (response rate%)	Exposure type and assessment	Outcome type and assessment	Confounders considered in analyses	Findings	Bias Risk*
**Bartels, S et al., 2018** [18]	Ger	CS	1200 (34)	Air traffic noise recorded for every participant /dwelling	Annoyance	Type of flight, altitude Noise sensitivity, Attitudes, Urbanisation Age gender education	Weak association between noise estimates (17% variance explained)	Low
**Cho, Y et al., 2014** [19]	S. Kor	CS	381 (99)	Aircraft noise Modelled exposure levels expressed in Lden and measured	Annoyance (mean)	Length of residence, age gender	Lden related to mean annoyance levels (.45)Peak level gave a slightly better prediction than Lden Variability important component in prediction of annoyance	Medium
**Quehl, J et al., 2017** [20]	Ger	CS (Field study)	157 (eligible)	Aircraft Noise Recorded continuously inside the bedroom at the sleeper's ear.	Annoyance (acute and long-term)	Age gender, perceived loudness, noise sensitivity , long term annoyance, chronotype	Laeq seq short term High?)annoyance: OR = 1.090 (95% CI:1.047 1.143) Number of overflights: OR= 1.060 (95% CI 1.036 1.089)	Medium

Table S1.3 Overview of the characteristics of the selected studies on the association between rail traffic noise and annoyance.

Pub.	Country*	Design†	Sample size (response rate%)	Exposure type and assessment	Outcome type and assessment	Confounders considered in analyses	Findings	Bias Risk*
**Licitra, Get al., 2016** [21]	Ita	CS	119 Respons e rate unknown	Rail traffic (Modelled versus measured)	Annoyance (11 and 5 points scale)	Vibration	Average increase of 3 points of %HA at the same noise levels resulted between the	Medium

Pub.	Country[*]	Design[†]	Sample size (response rate%)	Exposure type and assessment	Outcome type and assessment	Confounders considered in analyses	Findings	Bias Risk*
							simulated and measured values, which include the unconventional sources. (very different from Miedema generalized curve)	
**Pennig, S et al., 2014** [22]	Ger	CS	320 (22)	Railway An acoustical simulation model for this area calculated individual noise exposure levels.	Annoyance	Worry, coping, control , noise sensitivity	60% HA which is extremely high compared to German federal findings (3%HA) EEr compared to Miedema curve also much higher levels	Low

Table S1.4 Overview of the characteristics of the selected studies on the association between wind turbine noise and annoyance.

Pub.	Country*	Design†	Sample size (response rate%)	Exposure type and assessment	Outcome type and assessment	Confounders considered in analyses	Findings	Bias Risk*
**Michaud, D et al, 2016** [23]	Ca	CS	1238 (79)	Wind turbines A waited SPL outdoors estimated + C weighted	Annoyance	Age, gender, education, lifestyle, chronic illness , stress, WHOqol, dwelling characteristics shadow flickering etc.	Increase in Percentage high annoyance with increasing A-weighted levels R2= 9 % OR 2.38 (1.42, 3.99)	Low
**Klæboe, R et al., 2016** [24]	Nor	CS (after) study	90(38)	Wind turbines calculations range between 37-47	Annoyance (ISO 5 point scale)	Attitudes, demographics visual judgements, noise sensitivity	Effect from wind turbines visible at lower level (18 dB) than road traffic noise– when we disregard the large impact on annoyance from non-acoustic factors. The difference disappears when non-acoustical factors are accounted for	Medium
**Pawlaczyk - Łuszczyńska, M et al., 2014** [25]	Pol	CS	361 Response rate unknown	Wind turbine Calculated and measured in situ at	Annoyance	Attitude, visual aspects age gender education	Significant association between level of	Medium

Pub.	Country[*]	Design[†]	Sample size (response rate%)	Exposure type and assessment	Outcome type and assessment	Confounders considered in analyses	Findings	Bias Risk*
				selected addresses		type of house,	noise and annoyance Exp(b) = 2.16,(c)	
**Pawlaczyk - Łuszczyńska, M et al., 2014b [26]**	Pol	CS (pilot study)	156 Response rate unknown	Wind turbine Calculated levels and measurements	Annoyance (ISO 5 point scale)	Attitude, visual aspects age gender education type of house,	Wind turbine noise SPLs associated with increased percentage highly annoyed (OR = 2.1; 95% CI: 1.22–3.62)	Medium
**Pawlaczyk - Łuszczyńska, Met al., 2018 [27]**	Pol	CS	517 (78)	Wind turbines Calculated levels and randomly verified by in situ measurement	Annoyance (ISO 5 point scale)	Satisfaction, visual aspects, demographics, attitude	Annoyance, High annoyance increase with increase in SPl (OR > 1.00), negative attitude towards wind turbines, and decreased with an increasing distance from the nearest wind turbine (OR < 1.00),	Medium

Table S1.5 Overview of the characteristics of the selected studies on the association between combined sources and annoyance.

Pub.	Country*	Design†	Sample size (response rate%)	Exposure type and assessment	Outcome type and assessment	Confounders considered in analyses	Findings	Bias Risk*
**Brink M, et al., 2019** [28]	Swi	CS	5592 (31)	Road, Rail, Air traffic) Laeq , Lden and Intermittency ratio	Annoyance	Intermittent noise demographics, seasonal differences,	Sign. association for all sources and all outcomes but highest for road traffic noise	Low
**Vianna, K.M.D. P et al., 2015** [29]	Por	CS	180 (65-75)	Urban soundscapes at home, recreational, work Noise maps at façade expressed in Lden Exposed-non exposed.	Annoyance per sources (self-reported, 3 point scale	Demographic characteristics, sex, age and marital status; type of noise (related to activity)	Lden related to % of annoyed, highly annoyed in three different scenario's (activity is included as co-variate)	Medium
**Nguyen et, T.L al., 2016** [30]	JP	Series of CS studies	9900 Response rates 85, 74 (road, air)	Road, Air (Measurements)	Annoyance	Demographics,	Vietnamese respondents were less annoyed by road traffic noise than respondents in the European &Korean studies. Aircraft noise annoyance curve for Vietnam was slightly higher than that for the EU, but considerably lower than that in the Korean study.	Medium
**Sung et, J.H. al., 2016** [31]	S.Kor	CS	1000 (43%) 1000 (51%)	Road and Air day-night equivalent sound level	Annoyance	Age, gender, residence duration, income, marital	Increase %AH 9.0% <55 dBA group, to 11.5% and	Medium

Pub.	Country*	Design†	Sample size (response rate%)	Exposure type and assessment	Outcome type and assessment	Confounders considered in analyses	Findings	Bias Risk*
			Total 1836 (after selection)	(Ldn). Categorized into 3 levels.		status, lifestyle	17.3% in the 55±65 dBA and greater than 65 dBA groups, respectively ($p<0.001$). OR2.056 (95% [CI] 1.225±3.450), 3.519 (95% CI 1.982±6.246) in Seoul and 1.022 (95% CI 0.585±1.785), 1.704 (95% CI1.005±2.889) in Ulsan, respectively	

Table S1.6 Overview of the characteristics of the selected studies on the association between road traffic noise and sleep disturbance.

Pub.	Country*	Design†	Sample size (response rate%)	Exposure type and assessment	Outcome type and assessment	Confounders considered in analyses	Findings	Bias Risk*
**Evandt, J et al., 2016**[32]	Nor	CS	13019 (48)	Road traffic noise Night time Modelled at façade Lnight, A-weighted night time)	Sleep disturbance (self-reported)	age, sex, marital status, alcohol use, smoking, physical activity, and night-shift work, socioeconomic status Noise sensitivity, Chronic disease	Difficulties falling asleep (OR) 1.05 (95% confidence interval [CI]: 1.01–1.09) Awakenings during the night, OR 1.04 (95% CI: 1.00–1.08) Waking up too early, OR 1.06 (95% CI: 1.02–1.11).	Low
**Han, Z.-X et al., 2014** [33]	Chi	CS	400 (from 4 areas) - Residential-Construction - Transportation hub - Commercial	Road traffic/transport Noise measurements (monitoring, at different moments) 78 dBA , 71 at night	Sleep quality (self-reported) measured by the Pittsburg Sleep Index	age, sex, and educational level.	Sleep quality lowest I transportation hub $Chi^{2}=$ 11.556 .(009) With 65% low sleep quality (versus 47% in the other areas)	High
**Joost, S. et al., 2018** [34]	Swi	CS (Within Cohort)	3697 (73) + 10% excluded	Road traffic noise Night time (Modelled)at 10x10 grid level	Daytime Sleepiness (self-reported)	BMI, neighbourhood level income. Gender, age, beta-blockers, antihypertensive drugs	Weak association with levels, stronger when spatial distribution was accounted for.	Medium
**Martens, A.L et al., 2018** [35]	NL	CS (within a	14929, (16) 7905 at follow up (54)	Road traffic modelled at address level	Sleep quality	Age gender smoking	β (95%CI) 0.05 (0.01,0.09) 0.008	Medium

Pub.	Country*	Design†	Sample size (response rate%)	Exposure type and assessment	Outcome type and assessment	Confounders considered in analyses	Findings	Bias Risk*
		cohort)		(Stamina) expressed in Lden	(self-reported)			

Table S1. 7 Overview of the characteristics of the selected studies on the association between air traffic noise and sleep disturbance.

Pub.	Country*	Design†	Sample size (response rate%)	Exposure type and assessment	Outcome type and assessment	Confounders considered in analyses	Findings	Bias Risk*
**Holt, J.B. et al., 2015**[36]	US	CS (Surveillance data)	745,868 (88)	Aircraft Modelled noise levels	Sleep disturbance (self-reported)	Age, gender, race/ethnicity educational level, smoking and obesity,	No significant associations between airport noise and sleep insufficiency.	Low
**Kim, K. et al., 2019** [37]	S Kor	CS	1005(47)	Aircraft Modelled High, Low Control	Sleep quality (self-reported)	Mental health, age, gender, residence duration	Firstly, the prevalence of sleep disturbance significantly differed according to the noise level (p for trend < 0.001).	Medium
**Kwak, K. et al., 2016** [38]	S.Kor	CS	3308 Response rate unknown	Aircraft High, Low and No exposure groups based on modelled noise level	Sleep disturbance (self-reported) Insomnia Index and Epworth sleepiness scale (self-reported)	Age, gender, education, lifestyle, hospital, smoking, drinking, physical activity, length of residence	The risk of insomnia was 3.45 times (95 % CI 2.64-4.50) higher in the low exposure group and 3.24 times (95 % CI 2.48-4.22) higher in the high exposure group, as compared to that of	Medium

Pub.	Country*	Design†	Sample size (response rate%)	Exposure type and assessment	Outcome type and assessment	Confounders considered in analyses	Findings	Bias Risk*
							the control group. The risk of insomnia was 3.41 times (95% CI 2.61-4.46) higher in the low exposure group and 3.26 times (95% CI 2.50- 4.25) in the high exposure group after adjustment for confounders	
**Nassur, A.-M. et al., 2017** [39]	Fr	CS	1,244 (30)	Aircraft noise (modelled) Noise maps	Sleep time and tiredness next day (self-reported)	Demographics, lifestyle, SES	OR of 1.63 (95% CI: 1.15–2.32) for a short sleep time OR of 1.23 (95% CI: 1.00–1.54) for the feeling of tiredness next day	Low
**Nassur, A.-M. et al., 2019** [42]	Fr	CS (Field study)	112 volunteers selected from 1244 (30)	Aircraft noise Measured noise levels indoor during night	Sleep (Actigraphics)	Age; gender; marital status; education; and body mass index (BMI).	Increased levels of aircraft noise and increased numbers of aircraft noise events increased the time required for sleep onset (SOL) and the total wake time after sleep onset	Low

Pub.	Country*	Design†	Sample size (response rate%)	Exposure type and assessment	Outcome type and assessment	Confounders considered in analyses	Findings	Bias Risk*
							(WASO) and decreased sleep efficiency (SE). increase in total sleep time (TST) and time in bed (TB).with OR range of 1.10-1.60	

Table S1.8 Overview of the characteristics of the selected studies on the association between wind turbine noise and sleep disturbance.

Pub.	Country*	Design†	Sample size (response rate%)	Exposure type and assessment	Outcome type and assessment	Confounders considered in analyses	Findings	Bias Risk*
**Kageyama , T. et al., 2016** [40]	JP	CS	1079 (47)	Wind turbines Field measurements during the survey estimates per address 36-40 dB and <35	Sleep symptoms (self-reported) Insomnia (self-reported)	Road traffic Noise sensitivity Attitudes wind turbine Age gender, education	Insomnia more prevalent in areas with levels > 40 at night, But on 1.2 was defined as Insomniac Note also more women participated (52% and 61 in the control group)	Medium
**Michaud, D. et al., 2016** [41]	Ca	CS	1238 (79)	Wind turbines A waited SPL outdoors estimated + C weighted	Sleep disturbance (self-reported)	Age, gender, education, lifestyle, chronic illness , stress, WHOqol, dwelling characteristics shadow flickering annoyance	No effect on any of the sleep indicators	Low
**Michaud, D. et al., 2016** [41]	Ca	CS	742 (subsample)	Wind turbines A waited SPL outdoors estimated + C weighted	Sleep (Actigraphics)	Age, gender, education, lifestyle, chronic illness , stress, WHOqol, dwelling characteristics shadow flickering annoyance	No effect on any of the sleep indicators	Low
**Poulsen , A.H. et al., 2019** [43]	Den	CS	583,968 addresses after exclusion of people who emigrated etc	Wind turbines Modelled and > 24 db Outdoor and LFN indoor (10-160 Hz)	Sleep (Prescribed medication)	Age, gender, income, education, marital status Dwelling, distance to the road	Five-year mean outdoor night time WTN of ≥42 dB was associated with a hazard ratio(HR)=1.14[95% CI]:0.98,	Medium

Pub.	Country*	Design†	Sample size (response rate%)	Exposure type and assessment	Outcome type and assessment	Confounders considered in analyses	Findings	Bias Risk*
							1.33) for sleep medication Indoor: Nighttime LFWTN,the HRs(95%CIs)among persons ≥65 exposed to ≥15 dB were1.37(95% CI:0.81,2.31) for sleep medication	

Table S1.9 Overview of the characteristics of the selected studies on the association between mixed noise sources and annoyance, sleep disturbance.

Pub.	Country*	Design†	Sample size (response rate%)	Exposure type and assessment	Outcome type and assessment	Confounders considered in analyses	Findings	Bias Risk*
**Perron, S et al., 2016** [44]	Ca	CS	4336 Response rate unknown	Road Rail Air noise Night for each study participant was estimated using a land use regression	Sleep disturbance (self-reported)	Noise sensitivity age gender	Percentage of people sleep disturbed by road traffic, airplane and railway noise was 4.2%, 1.5% and 1.1% respectively, respectively	Medium
**Paiva , K.M. et al., 2019** [45]	Bra	CS	225 Response rate unknown	Road (modelled)	Annoyance, Sleep disturbance (self-reported) (three point scale and dichotomous)	Demographics, year of residence etc. etc	Strong association but the scale was not properly used and the necessary statistics are not available.	Medium
**Carugno , M. et al., 2018** [46]	Ita	CS	400 (35)	Aircraft noise While adjusting for other sources Acoustic Zones 60-65, 65-77, > 75 Lden	Annoyance, Sleep disturbance (self-reported)	Demographics, drugs, clinical history, other noise sources , housing type, lifestyle occupation, BMI	Association /trend between zones and mean annoyance scores (range from 33, 66, and 80%). Zones A and B: more sleep disorders (awakenings, sleep onset, poor	Low

Pub.	Country*	Design†	Sample size (response rate%)	Exposure type and assessment	Outcome type and assessment	Confounders considered in analyses	Findings	Bias Risk*
							quality duration)	
**Pultznerova, A. et al., 2018** [47]	Slo	CS	107 (100)	Rail traffic measured and modelled (noise maps)	Annoyance, Sleep quality (self-reported)	Age, gender, type of home, type of work, floor level,	%HA OR 7.80 (4.02–15.14)*** Sleep Quality: OR 1.95 (95% CI 1.20–3.18), chi2= 7.31 (0.006)	Medium
**Radun, J et al., 2019** [48]	Fin	CS	429 (57) 318 eligible	Wind turbines Modelled levels And categorised [25–30],[30–35], [35–40), and [40–46] Lden	Annoyance, Sleep disturbance. (self-reported) (indoor, outdoor)	Trust in authorities and operators, visibility, economic benefits, age, gender, education, type of dwelling, distance	Sound level [dB] Annoyance outdoor 1.41 (1.14, 1.74) <0.01 .(R2= .71) Indoor none Sleep 1.38 (95% CI 1.16, 1.65) <0.01(R2= .50)	Low
**Song, K. et al., 2016** [49]	Chi	CS	227 (77)	Wind turbine Measurements, categorized into 5 noise levels (44.1 dBA to 56.7dBA)	Annoyance, Sleep disturbance (self-reported)	Gender, age residence time, visibility, noise sensitivity, attitude, general opinion about WTs	%HA increased from 39.5% (95% CI: 28.4–51.4%) to 75.0% (95% CI: 50.9–91.3% Sleep disturbance and LAeq Spearman correlation= 0.209	Medium

Pub.	Country*	Design†	Sample size (response rate%)	Exposure type and assessment	Outcome type and assessment	Confounders considered in analyses
Argalášová, L. et al., 2014 50	Pol	CS Longitudinal	511 (1989)<br>857 (1999)<br>808 (2004)<br>932 (2013)<br>(90)	Road, Air Measurements, categorized in exposed and controls	Annoyance, Sleep disturbance (self-reported)	Age gender smoking alcohol, type of building, quiet side
Douglas, O. et al., 2016 51	Ire	CS	208(90)	Road, Rail Air Random measurements LAeq, LA90, and LAmax. Excluded families with children and with other noise sources	Annoyance, Sleep disturbance (self-reported)	Gender, and social class together with dwelling information., building age, double glazing
Bodin, T. et al., 2015 52	Swe	CS	2612 (54)	Rail and road traffic noise Modelled	Annoyance Sleep disturbance (self-reported)	Access quiet side, window facing yard, age, gender, smoking, hearing, bmi, noise sensitivity

[1] *In Segment 1 results are presented per publication instead of study (cardiovascular and metabolic effects per study/only the new publications are included)*

* Country: Bra=Brazil; Ca= Canada; Chin= China, Ind = India; Ire = Ireand; JP=japan; Tha=Thailand; Slo=Slovakia; Sp=Spain; Swi=Switzerland; UK = United Kingdom; US=United States of America; Den = Denmark; Gre = Greece, Nor = Norway; Swe = Sweden, Fin=Finland; Ger = Germany; NL = The Netherlands; Fr=France S.Kor=South Korea; Ita=Italy; Pol=Poland; Por = Portugal

†Design: CS = Cross-sectional study, CO = Cohort study, ‡ The number of people (N) and the response rate (in case of a cross-sectional study) (%);

Segment 2

Table S2. 1 Overview of the characteristics of the selected studies on the association between aircraft noise and hypertension.

Study	Country*	Design†	Study population			Exposure range (dB) in LDEN	Ascertainment hypertension** (prev/inc/mor)	Status††
			N (%)‡	Sex#	Age range (yrs)			
HYENA-Gr [53]	Gre	CO	420 (46)	MF	45-70	35-40, 40-45, 45-50, 50-55, 55-60, ≥60[a]	1, 2 (inc)	1
SDPP [54]	Swe	CO	4,854	MF	35-56	<50, 50-54, 55-59, ≥60	1, 2 (inc)	2
NOrAH [57-59]	Ger	CC	493,168	MF	≥40	<40, 40-45, 45-50, 50-55, 55-60, ≥60	3 (inc)	1

Table S2.2 Overview of the characteristics of the selected studies on the association between road traffic noise and hypertension.

Study	Country*	Design†	Study population			Exposure range (dB) in LDEN	Ascertainment hypertension** (prev/inc/mor)	Status††
			N (%)‡	Sex#	Age range (yrs)			
DCH [65]	Den	CO	24,181	MF	50-64	~ 55 – 70	2 (inc)	1
HYENA-Gr [53]	Gre	CO	420 (46)	MF	45-70	<30, 30-35, 35-40,40-45, 45-50, 50-55, 55-60, ≥60	1, 2 (inc)	1
HUBRO[63]	Nor	CO	4,462	MF	22-75	~30-75	2 (inc)	1
SNAC-K [63]	Swe	CO	1,945	MF	≥60	~55-75	1, 2 (inc)	2
HNR [63]	Ger	CO	4,507	MF	45-75	~35-70	1, 2 (inc)	1
KORA [63, 64]	Ger	CO	5,177	MF	25-74	~45 – 65	1, 2 (inc)	2
REGICOR [63]	Sp	CO	1,931	MF	36-82	~60 – 75	1, 2 (inc)	1
SDPP [54]	Swe	CO	4,854	MF	35-54	<45, 45-49, 50-54, ≥55	1, 2 (prev)	2
NORAH [57-59]	Ger	CC	493,168	MF	≥40	<40, 40-45, 45-50, 50-55, 55-60, 60-65, 65-70, ≥70	1, 2 (prev)	2

Table S2.3 Overview of the characteristics of the selected studies on the association between rail traffic noise and hypertension.

Study	Country*	Design†	Study population			Exposure range (dB) in L_{DEN}	Ascertainment hypertension** (prev/inc/mor)	Status††
			N (%)‡	Sex#	Age range (yrs)			
SDPP [54]	Swe	CO	4,854	MF	35-55	<45, 45-49, 50-54, ≥55	1, 2 (inc)	1
NORAH [57-59]	Ger	CC	493,168	MF	≥40	<40, 40-45, 45-50, 50-55, 55-60, 60-65, 65-70, ≥70	3 (inc)	1

Table S2.4 Overview of the characteristics of the selected studies on the association between wind turbine noise and hypertension.

Study	Country*	Design†	Study population			Exposure range (dB) in WTN	Ascertainment hypertension** (prev/inc/mor)	Status††
			N (%)‡	Sex#	Age range (yrs)			
DWS [71, 72]	Den	CO	535,675	MF	25-85	<24, 24-30, 30-36, 36-42, ≥42	3 (inc)	1

Table S2.5 Overview of the characteristics of the selected studies on the association between aircraft noise and ischemic heart disease.

Study	Country*	Design†	Study population			Exposure range in LDEN	Ascertainment IHD** (prev/inc/mor)	Status††
			N (%)‡	Sex#	Age range (yrs)			
HYENA_GRE [53]	Gre	CO	420	MF	45-70	<30, 30-35, 35-40,40-45, 45-50, 50-55, 55-60, ≥60	2 (inc)	1
SNC [73, 74]	Swi	CO	4,404,046	MF	>30	≤30, 30-40, 40-50, 50-60, ≥60	3 (mor)	2
NORAH [58, 77]	Ger	CC	854,366	MF	≥40	<40, 40-45, 45-50, 50-55, 55-60, ≥60	3 (inc, mor)	1
CAENS [78]	Swe	CO	20,012	MF	≥35	<45, 45-50, 50-55, ≥55	3 (inc)	1

*

Table S2.6 Overview of the characteristics of the selected studies on the association between road traffic noise and ischemic heart disease.

Study	Country*	Design†	Study population			Exposure range in LDEN	Ascertainment IHD** (prev/inc/mor)	Status††
			N (%)‡	Sex#	Age range (yrs)			
DCH [79]	Den	CO	50,744	MF	50-64	~48-72	3 (inc)	2
SPHC [80]	Swe	CO	9,031	MF	18-80	<45, 45-55, 55-65, 65-80	3 (inc)	1
HUNT2 [81, 82]	Nor	CO	43,267	MF	≥20	~42-70	3 (inc)	1
EPIC-Oxford [81, 82]	UK	CO	23,909	MF	≥20	~51-85	3 (inc)	1
UK-Biobank [81, 82]	UK	CO	288,556	MF	40-69	~51-87	3 (inc)	1
HYENA_GRE [53]	Gre	CO	420	MF	45-70	<30, 30-35, 35-40,40-45, 45-50, 50-55, 55-60, ≥60	2 (inc)	1
CAENS [78]	Swe	CO	20,012	MF	≥35	<45, 45-50, 50-55, ≥55	3 (inc)	1
HNR [83]	Ger	CO	4,433	MF	45-74	~28-63[b]	1 (inc)	1
SNC [73, 74]	Swi	CO	4,404,046	MF	≥30	≤45, 45-50, 50-55, 55-60, 60-65, >65	3 (mor)	1
NORAH [58, 77]	Ger	CC	854,366	MF	≥40	<40, 40-45, 45-50, 50-55, 55-60, ≥60	3 (inc, mor)	2

Table S2.7 Overview of the characteristics of the selected studies on the association between rail traffic noise and ischemic heart disease.

Study	Country*	Design†	Study population			Exposure range in LDEN	Ascertainment IHD** (prev/inc/mor)	Status††
			N (%)‡	Sex#	Age range (yrs)			
**SNC [73, 74]**	**Swi**	**CO**	**4,404,046**	**MF**	≥30	≤30, 30-40, 40-50, 50-60, >60	3 (mor)	1
NORAH[58,77]	Ger	CC	854,366	MF	≥40	<40, 40-45, 45-50, 50-55, 55-60, ≥60	3 (inc, mor)	1
CAENS [78]	Swe	CO	20,012	MF	≥35	<45, 45-50, 50-55, ≥55	3 (inc)	1

Table S2.8 Overview of the characteristics of the selected studies on the association between wind turbine noise and ischemic heart disease.

Study	Country*	Design†	Study population			Exposure range (dB) in LDEN	Ascertainment IHD** (prev/inc/mor)	Status††
			N (%)‡	Sex#	Age range (yrs)			
**DNC[85]**	**Den**	**CO**	**23,994**	**F**	≥44	Unexposed, <21.5, 21.5-25.4, 25.4-29.9, >29.9	3 (inc)	1
DWS[72, 86]	Den	CO	535,675	MF	25-85	<24, 24-30, 30-36, 36-42, ≥42	3 (inc)	1

Table S2.9 Overview of the characteristics of the selected studies on the association between air traffic noise and stroke.

Study	Country*	Design†	Study population			Exposure range in LDEN	Ascertainment stroke** (prev/inc/mor)	Status††
			N (%)‡	Sex#	Age range (yrs)			
**SNC** [74]	**Swi**	**CO**	**4,415,206**	**MF**	≥30	<30, 30-40, 40-50, 50-60, >60	3 (mor)	2
HYENA_GRE [53]	Gre	CO	420	MF	45-70	<30, 30-35, 35-40,40-45, 45-50, 50-55, 55-60, ≥60	2 (inc)	1
NORAH [58, 87]	Ger	CC	853,096	MF	≥40	40-45, 45-50, 50-55, 55-60, 60-65, 65-70, ≥70	3 (inc, mor)	1
CAENS [78]	Swe	CO	20,012	MF	≥35	<45, 45-50, 50-55, ≥55	3 (inc)	1

Table S2.10 Overview of the characteristics of the selected studies on the association between road traffic noise and stroke.

Study	Country*	Design†	Study population			Exposure range in LDEN	Ascertainment stroke** (prev/inc/mor)	Status††
			N (%)‡	Sex#	Age range (yrs)			
**HYENA_GRE** [53]	**Gre**	**CO**	**420**	**MF**	45-70	<30, 30-35, 35-40,40-45, 45-50, 50-55, 55-60, ≥60	2 (inc)	1
HUNT2 [81, 82]	Nor	CO	43,267	MF	≥20	~42-70	3 (inc)	1
EPIC-Oxford [81, 82]	UK	CO	23,909	MF	≥20	~51-85	3 (inc)	1
UK-Biobank [81, 82]	UK	CO	288,556	MF	40-69	~51-87	3 (inc)	1
HNR [83]	Ger	CO	4,433	MF	45-74	~28-63e	1 (inc)	1
SNC [74]	Swi	CO	4,415,206	MF	≥30	<30, 30-40, 40-50, 50-60, >60	3 (mor)	1
NORAH [58, 87]	Ger	CC	853,096	MF	≥40	40-45, 45-50, 50-55, 55-60, 60-65, 65-70, ≥70	3 (inc, mor)	1
CAENS [78]	Swe	CO	20,012	MF	≥35	<45, 45-50, 50-55, ≥55	3 (inc)	1

Table S2.11 Overview of the characteristics of the selected studies on the association between rail traffic noise and stroke.

Study	Country*	Design†	Study population			Exposure range in LDEN	Ascertainment stroke** (prev/inc/mor)	Status††
			N (%)‡	Sex#	Age range (yrs)			

SNC [74]	Swi	CO	4,415,206	MF	≥30	<30, 30-40, 40-50, 50-60, >60	3 (mor)	1
NORAH [58, 87]	Ger	CC	853,096	MF	≥40	40-45, 45-50, 50-55, 55-60, 60-65, 65-70, ≥70	3 (inc, mor)	1
CAENS [78]	Swe	CO	20,012	MF	≥35	<45, 45-50, 50-55, ≥55	3 (inc)	1

Table S2.12 Overview of the characteristics of the selected studies on the association between aircraft noise and diabetes.

Study	Country[*]	Design[†]	Study population			Exposure range (dB) in LDEN	Ascertainment Diabetes[**] (prev/inc/mor)	Status[††]
			N (%)[‡]	Sex[#]	Age range (yrs)			
HYENA_GRE [53]	Gre	CO	420 (78)	MF	45-75	<30, 30-35, 35-40,40-45, 45-50, 50-55, 55-60, ≥60	1 (inc)	1
SAPALDIA [88]	Swi	CO	2,631	MF	Adults	<50, 50-55, >55	1, 2 (inc)	1

Table S2.13 Overview of the characteristics of the selected studies on the association between road traffic noise and diabetes.

Study	Country[*]	Design[†]	Study population			Exposure range (dB) in LDEN	Ascertainment diabetes[**] (prev/inc/mor)	Status[††]
			N (%)[‡]	Sex[#]	Age range (yrs)			
DCH [89]	Den	CO	50,534	MF	50-64	~48-71	3 (inc)	2
HYENA_GRE [53]	Gre	CO	420 (78)	MF	45-75	<30, 30-35, 35-40,40-45, 45-50, 50-55, 55-60, ≥60	1 (inc)	1
SAPALDIA [88]	Swi	CO	2,631	MF	Adults	<50, 50-55, >55	1, 2 (inc)	1

Table S2.14 Overview of the characteristics of the selected studies on the association between rail traffic noise and diabetes.

Study	Country*	Design†	Study population			Exposure range (dB) in LDEN	Ascertainment diabetes** (prev/inc/mor)	Status††
			N (%)‡	Sex#	Age range (yrs)			
DCH [89]	Den	CO	50,534	MF	50-64	~ 20-80	3 (inc)	2
SAPALDIA [88]	Swi	CO	2,631	MF	Adults	<50, 50-55, >55	1, 2 (inc)	1

Table S2.15 Overview of the characteristics of the selected studies on the association between wind turbine noise and diabetes.

Study	Country*	Design†	Study population			Exposure range (dB) in LDEN	Ascertainment Diabetes** (prev/inc/mor)	Status††
			N (%)‡	Sex#	Age range (yrs)			
DWS [72, 92]	Den	CO	614,731	MF	25-85	< 24, 24–<30, 30–<36, 36–<42, ≥42	3 (inc)	1

Table S2.16 Overview of the characteristics of the selected studies on the association between aircraft noise and indicators of obesity.

Study	Country*	Design†	Study population			Exposure range (dB) in LDEN	Indicator of obesity**	Status††
			N (%)‡	Sex#	Age range (yrs)			
SAPALDIA-b [93]	Swi	CO	3,796 (83)	MF	18-60	~30 – 58	CO, OW, BMI	1
SDPP [94]	Swe	CO	5,184 (91)	MF	35-55	<45, 45-49,50-54, ≥55	BMI, WC, WG, CO, OW	2

Table S2.17 Overview of the characteristics of the selected studies on the association between road traffic noise and indicators of obesity.

Study	Country*	Design†	Study population			Exposure range (dB) in LDEN	Indicator of obesity**	Status††
			N (%)‡	Sex#	Age range (yrs)			
SDPP [94]	Swe	CO	5,184 (91)	MF	35-54	<45, 45-49,50-54, ≥55	BMI, WC, WG, CO, OW	2
DCH[95]	Den	CO	39,720	MF	50-64	<55, 55-60, 60-65, >65	BMI, WG, WC	2
SAPALDIA-b [93]	Swi	CO	3,796 (83)	MF	18-60	~35-75	CO, OW, BMI	1

Table S2.18 Overview of the characteristics of the selected studies on the association between rail traffic noise and obesity.

Study	Country[*]	Design[†]	Study population N (%)[‡]	Sex[#]	Age range (yrs)	Exposure range (dB) in LDEN	Indicator of obesity[**]	Status[††]
SDPP [94]	Swe	CO	5,184 (91)	MF	35-54	<45, 45-49,50-54, ≥55	BMI, WC, WG, CO, OW	2
DCH [95]	Den	CO	39,720	MF	50-64	<55, 55-60, 60-65, >65	BMI, WG, WC	2
SAPALDIA-b [93]	Swi	CO	3,796 (83)	MF	18-60	~30-75	CO, OW, BMI	1

Legend

Chin= China, Ind = India, Sp = Spain, UK = United Kingdom, Den = Denmark, Gre = Greece, Nor = Norway, Swe = Sweden, Ger = Germany, NL = The Netherlands; †Design: ECO = Ecological study, CS = Cross-sectional study, CO = Cohort study, CC = Case control study; ‡ The number of people (N) and the response rate (in case of a cross-sectional study) (%); # M = Men, F = Females;

** The way hypertension was ascertained: 1 = measurement of blood pressure levels and/or by means of a clinical interview, 2 = by means of a question as part of a questionnaire or interview (self-reported), 3 = by means of health registration database.

** The way stroke was ascertained: 1 = by means of a clinical interview/anamnesis, 2 = by means of a question as part of a questionnaire or interview (self-reported), 3 = by means of health registration database.

** Indicator of obesity: BMI = change in Body Mass Index (kg/m2), BF = percentage body fat, WC = Change in waist circumference (cm/yr), WG = Weight gain (kg/yr), CO = incidence of Central Obesity, OW = incidence of Overweight; BF = change in percentage body fat;

** The way diabetes was ascertained: 1 = measurement/clinical interview, 2 = self-reported, 3 = healthcare registration.

Type of outcome: prev = prevalence, inc = incidence, mor = mortality. †† 1 = Study identified and selected as part of the new literature search, 2 = Study already identified and selected as part of the WHO evidence review. Additional or new study results identified as part of the new literature search; a Noise exposure level expressed in LAeq7-21hr; b Noise exposure level expressed in Lnight; c Noise exposure level expressed in LAeq7-23hr

a This cohort comprises of respondents from four Swedish cohorts: The Stockholm Diabetes Preventive Program (SDPP), the SIXTY subcohort, the Screening Across the Lifetime Twin Study (SALTS) and the Swedish National Study on Aging and Care in Kungsholmen (SNAC-K).

Supplementary 3: Glossary

DR	dose-response
CI	Confidence interval
DALY	Disability-adjusted life year
DEN	Day-evening-night equivalent level
DW	Disability weight
EBoDe	Environmental Burden of Disease in the European Region
EBD	Environmental Burden of Disease
EEA	European Environment Agency
END	Environmental noise directive (2002/49/EC)
EEr	Exposure Effect Relation
ERR	Exposure Response Relation
ERF	Exposure Response Function
HIA	Health Impact Assessment
EU	European Union
HA	Highly annoyed people
HSD	Highly sleep disturbed people
Incidence	Measure of the probability of occurrence of a given medical condition in a population within a specific period of time
LAeq,th or Leq,th	A-weighted equivalent sound pressure level over (t) hours
L_{den}	Day-evening-night equivalent sound level
L_{dn}	Day-night equivalent sound level
L_{night}	Night equivalent sound level
Morbidity	the rate of disease in a population.
Mortality	A measure of the number of deaths in a given population
NAP	Noise action plan
NafP	Number of affected people
OR	Odds ratio
Prevalence	Actual number of cases of disease or injury present in a population at any particular moment in time.
PSG	Polysomnography
REM	Rapid eye movement (sleep stage)
RR	Relative Risk
SD	Standard deviation
SWS	Slow wave sleep
WHO	World Health Organization

[1] Reasons for exclusion [technical; other population/other outcome; other study design; review/background/other /irrelevant exposures]
